# Supplementary material for: Diabetes Mellitus screening and associated factors in Peru: A cross-sectional analysis of a national health survey
Source: PLOS Glob Public Health. 2025 Sep 3;5(9):e0005150. doi: 10.1371/journal.pgph.0005150 (PMC12407462; doi:10.1371/journal.pgph.0005150)
Supplement: S1 Table — (DOCX) [file pgph.0005150.s003.docx]

# Variable Definitions

# Demographic Variables

| Variable | Definition | Role in Study | Nature | Measurement Scale | Indicator in Survey | Categories |
| --- | --- | --- | --- | --- | --- | --- |
| Age | Age in completed years at the time of the interview. | Independent Variable | Continuous Quantitative | Nominal | Response to item QS23: completed years in the Health Survey. | - 18-34 years - 35 years or older |
| Sex | Biological condition distinguishing between male and female. | Independent Variable | Dichotomous Qualitative | Nominal | Record in item QSSEXO: in Health Survey. | - Male - Female |
| Area of Residence | Geographical area where the respondent's home is located. | Independent Variable | Dichotomous Qualitative | Nominal | Recorded in item HV025: Area of residence. | - Rural - Urban |
| Educational Level | Highest level of education completed by the respondent. | Independent Variable | Polytomous Qualitative | Nominal | Response to item HV109: What was the highest year or grade of education completed? | - No education - Incomplete Primary - Complete Primary - Incomplete Secondary - Complete Secondary - Higher Education |
| Ethnicity | Ancestry and customs with which the respondent identifies. | Independent Variable | Polytomous Qualitative | Nominal | Response to item QS25BB: Based on your ancestry and customs, do you consider yourself? | - Quechua - Aymara - Native Amazonian - Afro-Peruvian - White - Mestizo - Other Ethnicity |
| Wealth Index | Distribution of the population according to quintiles established by ENDES 2022. | Independent Variable | Polytomous Qualitative | Ordinal | Record in item HV270: Wealth Index. | - Poorest - Poor - Middle - Rich - Richest |
| Region of Origin | Natural Region of Peru where the respondent resides. | Independent Variable | Polytomous Qualitative | Nominal | Record in item SHREGION: Natural Region | - Metropolitan Lima - Rest of the Coast - Highlands - Jungle |
| Residence Altitude | Altitude in meters above sea level where the respondent resides. | Independent Variable | Continuous Quantitative | Nominal | Response to item: Altitude of the cluster in meters (variable HV040) from the Household Questionnaire. | - Less than 3000 m.a.s.l - 3000 m.a.s.l or higher |
| Health Insurance | Whether the respondent has any health insurance (ESSALUD, SIS, Armed Forces and Police). | Independent Variable | Dichotomous Qualitative | Nominal | Response to question QS26: Do you have health insurance? | - Yes - No |

# Clinical Variables

| Variable | Definition | Role in the Study | Nature | Measurement scale | Indicator in survey | Measurement |
| --- | --- | --- | --- | --- | --- | --- |
| T2DM Screening | Screening tests for T2DM recommended by the ADA, such as fasting plasma glucose, glycated hemoglobin measurement, or glucose tolerance test. | Dependent Variable | Qualitative | Nominal | Response to question QS107” “"In the past 12 months, has a doctor or other health professional measured your blood glucose or "sugar" level?". | - Screened - Not Screened |
| Hypertension | Cardiovascular disease characterized by a chronically sustained blood pressure above the normal value | Independent variable | Qualitative | Nominal | Response to question QS102: "Has a doctor ever diagnosed you with hypertension or high blood pressure?" (Health Questionnaire) | - Hypertensive - No Hypertension |
| Smoker | If the respondent smokes cigarettes daily. | Independent variable | Qualitative dichotomous | Nominal | Response to question QS202: "Do you smoke daily?" from the Health Questionnaire | - Yes - No |
| Current Alcohol Consumption | Current intake of alcoholic beverages such as beer, wine, among others, by the respondent. | Independent variable | Qualitative dichotomous | Nominal | Response to question QS210: "In the last 30 days, have you consumed any alcoholic beverage or liquor?" from the Health Questionnaire | - Present - Absent |
