# Supplementary material for: Diabetes Mellitus screening and associated factors in Peru: A cross-sectional analysis of a national health survey
Source: PLOS Glob Public Health. 2025 Sep 3;5(9):e0005150. doi: 10.1371/journal.pgph.0005150 (PMC12407462; doi:10.1371/journal.pgph.0005150)
Supplement: Attachment — Submitted filename: PGPH-D-25-00415_reviewer.pdf [file pgph.0005150.s004.pdf]

PLOS Global Public Health

Diabetes Mellitus screening and associated factors in Peru: a cross-sectional analysis of a national health survey

--Manuscript Draft--

**Manuscript Number:**	PGPH-D-25-00415
**Article Type:**	Research Article
**Full Title:**	Diabetes Mellitus screening and associated factors in Peru: a cross-sectional analysis of a national health survey
**Short Title:**	Diabetes Mellitus screening in Peru: cross-sectional analysis of a national health survey
**Corresponding Author:**	Einer Carlos Eduardo Arevalo Rios, M.D.<br>Universidad Peruana Cayetano Heredia Facultad de Medicina<br>Lima, PERU
**Order of Authors:**	Einer Carlos Eduardo Arevalo-Rios, M.D.
	Jennifer Layza-Reyes, M.D.
	Victor Hugo Noriega-Ruiz, M.D.
**Keywords:**	Diabetes Mellitus; screening; Peru
**Abstract:**	Screening rates for Type 2 diabetes mellitus (T2DM) in Peru remain low despite an increasing prevalence of the disease. In 2022, the American Diabetes Association (ADA) updated its screening recommendations, lowering the screening age to 35 years, and including overweight or obese individuals with risk factors. Despite these updated recommendations, the extent of T2DM screening in Peru and the factors influencing low screening rates remain poorly understood. We aimed to determine the T2DM screening prevalence in Peru and the factors associated with lack of screening in the population that meets ADA screening criteria. A cross-sectional, secondary data analysis was conducted based on the 2022 Peruvian National Demographic and Health Survey. Screening was defined as having blood glucose measured in the last year. Individuals aged 18-34 years overweight or obese and all individuals aged ≥ 35 years were included. Bivariate analysis was performed using the chi-squared test, and multivariate Poisson regression was used to estimate prevalence ratios (PRs), adjusting for potential confounders. Of 26,166 surveyed individuals, 25.3% were screened for T2DM. The factors most strongly associated with lack of screening were: age 18-34 years (aPR: 1.08; 95% CI: 1.05-1.11), having only elementary education (aPR: 1.18; 95% CI: 1.13-1.23), not having health insurance (aPR: 1.15; 95% CI: 1.12-1.18), belonging to the lowest wealth quintile (aPR: 1.09; 95% CI: 1.05-1.13), and daily smoking (aPR: 1.10; 95% CI: 1.01-1.19). The national rate of screening for T2DM in Peru is low (25.3%). There is a higher prevalence for a lack of screening in people who: are younger, have only primary education, do not have health insurance, belong to the lowest wealth quintile, and are smokers. Targeted interventions are needed to improve screening coverage in these high-risk populations.
**Additional Information:**	
**Question**	**Response**
**Financial Disclosure**<br><br>Enter a financial disclosure statement that describes the sources of funding for the work included in this submission and the role the funder(s) played. This includes grants and any commercial funding of the work or authors.	The author(s) received no specific funding for this work.

Powered by Editorial Manager® and ProduXion Manager® from Aries Systems Corporation

This statement will be typeset if the manuscript is accepted for publication.<br><br>*Please review the submission guidelines and the instructions link below for detailed requirements and guidance.*	
**Competing Interests**<br><br>On behalf of all authors, disclose any competing interests that could be perceived to bias this work.<br><br>This statement will be typeset if the manuscript is accepted for publication.<br><br>*Please review the instructions link below and PLOS Global Public Health's competing interests policy to determine what information must be disclosed at submission.*	The authors have declared that no competing interests exist.
**Data Availability**<br><br>Before publication, Authors are required to make fully available and without restriction all data underlying their findings. Please see our PLOS Data Policy page for detailed information on this policy.<br><br>A **Data Availability Statement**, detailing where the data can be accessed, is required at first submission. Insert your Data Availability Statement in the box below.<br><br>Please see the data reporting section of our submission guidelines for instructions on what you need to include in your Data Availability Statement.	We are submitting the raw data from our analysis in CSV format; these files are included as supporting information.

Powered by Editorial Manager® and ProduXion Manager® from Aries Systems Corporation

This statement will be typeset if the manuscript is accepted for publication.<br><br>*PLOS allows rare exemptions to address legal and ethical concerns. If you have legal or ethical restrictions, please use the box below to detail these in full sentences for the Journal team to consider.*	

Powered by Editorial Manager® and ProduXion Manager® from Aries Systems Corporation

Manuscript
Click here to access/download;Manuscript;JOURNAL Manuscript DM Screening Peru.docx

Diabetes Mellitus screening and associated factors in Peru: a cross-sectional analysis of a national health survey

Einer Carlos Eduardo Arevalo-Rios[1*], Jennifer Layza-Reyes [1], Victor Hugo Noriega-Ruiz [1,2]

[1] Alberto Hurtado School of Medicine, Universidad Peruana Cayetano Heredia, Lima, Peru

[2] Department of Endocrinology, Clinica Anglo Americana, Lima, Peru

* Corresponding author

E-mail: einer.arevalo@upch.pe (EA)

Abstract

Screening rates for Type 2 diabetes mellitus (T2DM) in Peru remain low despite an increasing prevalence of the disease. In 2022, the American Diabetes Association (ADA) updated its screening recommendations, lowering the screening age to 35 years, and including overweight or obese individuals with risk factors. Despite these updated recommendations, the extent of T2DM screening in Peru and the factors influencing low screening rates remain poorly understood. We aimed to determine the T2DM screening prevalence in Peru and the factors associated with lack of screening in the population that meets ADA screening criteria. A cross-sectional, secondary data analysis was conducted based on the 2022 Peruvian National Demographic and Health Survey. Screening was defined as having blood glucose measured in the last year. Individuals aged 18-34 years overweight or obese and all individuals aged ≥ 35 years were included. Bivariate analysis was performed using the chi-squared test, and multivariate Poisson regression was used to estimate prevalence ratios (PRs), adjusting for potential confounders. Of 26,166 surveyed individuals, 25.3% were screened for T2DM. The factors most strongly associated with lack of screening were: age 18-34 years (aPR: 1.08; 95% CI: 1.05-1.11), having only elementary education (aPR: 1.18; 95% CI: 1.13-1.23), not having health insurance (aPR: 1.15; 95% CI: 1.12-1.18), belonging to the lowest wealth quintile (aPR: 1.09; 95% CI: 1.05-1.13), and daily smoking (aPR: 1.10; 95% CI: 1.01-1.19). The national rate of screening for T2DM in Peru is low (25.3%). There is a higher prevalence for a lack of screening in people who: are younger, have only primary education, do not have health insurance, belong to the lowest wealth quintile, and are smokers. Targeted interventions are needed to improve screening coverage in these high-risk populations.

Introduction

Type 2 Diabetes Mellitus (T2DM) is one of the leading causes of mortality worldwide. However, early diagnosis and treatment can significantly reduce morbidity and mortality [1]. Longer periods without adequate metabolic control increase the risk of developing chronic complications that

worsen prognosis [1,2]. Screening with a fasting plasma glucose measurement, glycated hemoglobin (HbA1c), or glucose tolerance test is the main strategy for early detection of this disease. In 2022, the American Diabetes Association modified its screening recommendations to include all individuals without risk factors starting at age 35; and individuals over 18 years old with overweight or obesity who have one or more risk factors, including Latino as a high-risk race and ethnicity [3]. These modifications were made to improve sensitivity of screening and to promote early detection of disease. Following these recommendations, and considering Peruvians as Latinos, all individuals with overweight or obesity between the ages of 18 and 34, as well as everyone over 35, should be screened for T2DM in Peru.

Peru is considered an upper-middle-income country with a geographically and ethnically diverse population that has persistent barriers to healthcare access despite recent economic growth [4]. Its diverse topography is marked by differences in the three main natural regions of the country: coast, highlands and jungle. Approximately 58% of the population resides on the coast, with many of the healthcare facilities concentrated in the capital districts of each department. The highlands of Peru are inhabited by 28.1% of the population, with many of them living at high altitudes, with limited infrastructures, deficient road networks and poor access to health services. The jungle of Peru, comprising 13% of the population, has many scattered native-indigenous communities with limited interconnection and many of them only accessible by air or water [4]. These characteristics pose an intrinsic barrier to healthcare access throughout the nation that impact early disease detection, including T2DM screening. Regarding non-communicable diseases, the prevalence of T2DM in Peru has increased in the past decades, with latest reports estimating a prevalence of 5-7 % highlighting variations among regions in the nation (coast, highlands, and jungle) [5,6]. We hypothesize socioeconomic and cultural barriers affect the T2DM screening coverage. It is currently unknown how many people have been screened in the country following the latest ADA guideline recommendations. While the Ministry of Health in Peru has defined screening criteria in national guidelines, these have not been updated since 2016, despite the increasing prevalence of the disease in the country [5,7]. Since T2DM is a disease that leads to complications that can be prevented with timely diagnosis and treatment, we believe that the latest and more sensitive screening criteria should be prioritized, which are the ADA

screening recommendations. Therefore, in this study we used data from a nationally representative health survey to answer the following questions: What is the prevalence of diabetes screening in Peru, and what factors are associated with the lack of screening?

Materials and Methods

Study design

A cross-sectional secondary analysis was performed using the database of the 2022 National Demographic and Health Survey of Peru (ENDES in Spanish). This survey was conducted by the National Institute of Statistics and Informatics (INEI in Spanish) from January to December 2022 on a nationally representative sample. The ENDES has different questionnaires; in this study, information from the Health Survey was used, which collects data on non-communicable diseases, including T2DM. Information was collected through face-to-face interviews and anthropometric measurements were obtained by trained individuals. These data are accessible in the public domain at https://proyectos.inei.gob.pe/microdatos/ (S1-S2 Data).

The ENDES used a two-stage, probabilistic, balanced, stratified, and independent sampling design, covering all regions of Peru, in both urban and rural areas. The Health Survey was administered to 36 650 households and interviewed 31 917 individuals aged 15 years and over.

Study population

This study analyzed complete-case responses from individuals that met the 2022 American Diabetes Association (ADA) screening criteria, considering Latino/Hispanic ethnicity as a risk factor. Accordingly, the study included individuals aged 35 years or older, and those aged 18-34 years with overweight or obesity. Body Mass Indexes were calculated based on the anthropometric measures obtained in the interviews and as recorded in the Health Survey. Pregnant women, individuals with a previous diagnosis of T2DM and those taking medications for this disease were excluded.

Variables

The main dependent variable of this study was the self-report of diabetes mellitus screening; this was determined by the answer to the question: "In the past 12 months, has a doctor or other health professional measured your blood glucose or "sugar" level?". This outcome was categorized as a dichotomous variable: “screened” or “not screened”. Potential risk factors for the lack of screening were identified based on review of previous literature. These included age, sex, residence area, region of origin, altitude of residence, education level, ethnicity, wealth index and health insurance status. The wealth index was defined in quintiles as categorized in the National Health Survey and based on a cumulative measure of a household’s living standard. Clinical variables affecting screening rates in previous literature were identified and included in the analysis: previous hypertension diagnosis, current alcohol consumption, and smoking status. Age was further categorized in 18-34 and 35 years or older to reflect the distinct screening group recommendations of the ADA. Altitude of residence was further categorized into living at an altitude more than 3 000 meters above sea level (m.a.s.l.) or not, based on previous studies that have reported lower diabetes prevalence at higher altitude in Peru [8]. Further description of variables included in the survey is included as supplementary information (S1 Table).

Statistical analysis

Data was analyzed using STATA SE (18.0 version, College Station, Texas 77845, USA). To account for the complex sampling design of the ENDES and the analysis of a specific subpopulation, the *svy* and *subpop* commands were used. Raw databases were merged to include the sociodemographic, anthropometric and health questionnaire information of surveyed individuals. Categorical variables are reported as frequencies and proportions, while quantitative variables are reported as mean and standard error for normally distributed data. Chi-square tests were used for the bivariate analysis between the independent variables and the dependent variable. Multivariate analysis was conducted using generalized linear models with Robust Poisson regression variance estimation to calculate crude and adjusted prevalence ratios for non-screening, considering the expected outcome prevalence to be greater

than 10% [9]. The variables included in the multivariate model were the ones that showed association in the bivariate model and those epidemiologically relevant according to the literature review. A p-value ≤ 0.05 was considered statistically significant.

Ethics

Data from the ENDES survey is available publicly online as published by the National Institute of Statistics and Research in Peru (INEI). For this study, data was accessed on June 26th, 2024. The information collected did not allow for the identification of the surveyed individuals. As this is a secondary study and there was no contact with the study population, informed consent was not required. The protocol of this study was reviewed and approved by the Institutional Committee on Ethics in Human Research (CIEI) of the Peruvian University Cayetano Heredia in Lima, Peru.

Results

Study population

The subpopulation that met the ADA screening criteria (35 years or older and 18-34 years with overweight or obesity) was 26,166 individuals (Fig 1). Among them, 52.5% were women, 81.5% lived in urban areas, 33.4% had achieved higher education, and 83.2% had health insurance. The distribution of the socioeconomic status categories was homogeneous. 11.4% had been diagnosed with hypertension; 1.5% smoked daily, and 38.2% had consumed alcohol in the past 30 days. The full description of the sample characteristics is shown in Table 1.

Fig 1. Flowchart of participant selection

Table 1. Characteristics of the population that meets the ADA T2DM screening criteria

**Characteristics**	**Total** (N= 26 166)	**Weighted proportion** % (95 CI)
**Age (mean, SE)**	45.39 (0.18)	-
**Age (categorized)**		
18 – 34 years	7 251	27.7 (26.8 – 28.6)
35 or more years	18 915	72.3 (71.4 – 73.2)

**Sex**		
Male	12 440	47.5 (46.4 – 48.6)
Female	13 726	52.5 (51.4 – 53.6)
**Area of residence**		
Rural	4 830	18.5 (17.9 – 19.0)
Urban	21 336	81.5 (81.0 – 82.1)
**Education level**		
No education	1 045	4.0 (3.6 – 4.4)
Incomplete elementary	4 126	15.8 (15.0 – 16.6)
Complete elementary	1 473	5.6 (5.2 – 6.0)
Incomplete secondary	3 332	12.7 (12.0 – 13.5)
Complete secondary	7 456	28.5 (27.5 – 29.5)
Higher education	8 734	33.4 (32.3 – 34.5)
**Ethnicity**		
Native	7 335	28.0 (27.1 – 29.0)
Zambo, afro descendant	2 998	11.5 (10.8 – 12.2)
White	1 842	7.0 (6.5 – 7.6)
Mestizo	12 349	47.2 (46.1 – 48.3)
Other	327	1.3 (1.0 – 1.5)
Does not know	1 315	5.0 (4.6 – 5.5)
**Economic level**		
The poorest	4 839	18.5 (17.8 – 19.2)
Poor	5 216	19.9 (19.1 – 20.8)
Middle	5 555	21.2 (20.3 – 22.2)
Rich	5 422	20.7 (19.7 – 21.8)
The richest	5 133	19.6 (18.5 – 20.8)
**Region of origin**		
Metropolitan Lima	9 789	37.4 (36.3 – 38.6)
Rest of the coast	6 848	26.2 (25.2 – 27.2)
Highlands	6 363	24.3 (23.3 – 25.4)
Jungle	3 166	12.1 (11.4 – 12.8)
**Altitude**		
Less than 3 000 m.a.s.l.	22 291	85.2 (84.3 – 86.1)
Great or equal to 3 000 m.a.s.l.	3 876	14,8 (13.9 – 15.7)
**Health insurance**		
Yes	21 539	82.3 (81.4 – 83.2)
No	4 627	17.7 (16.8 – 18.6)
**Hypertension**		
Present	2 982	11.4 (10.7 – 12.1)
Absent	23 184	88.6 (87.8 – 89.2)
**Daily smoker**		
No	25 780	98.5 (98.2 – 98.8)
Yes	386	1.5 (1.2 – 1.8)
**Alcohol drinking**		
Present	16 182	61.8 (60.7 – 62.9)
Absent	9 984	38.2 (37.1 – 39.3)
**Glucose screening in the last 12 months**		
Yes	6 616	25.3 (24.4 – 26.2)
No	19 550	74.7 (73.8 – 75.6)

SE = Standard error, m.a.s.l. = meters above sea level

Prevalence of T2DM screening and associated factors

Among individuals who met the ADA T2DM screening criteria, only 25.3% had a blood sugar measurement in the last 12 months. Bivariate analysis (Table 2) revealed that screening was less common among the rural population (85.7%), individuals without formal education (80.5%) and those from lower socioeconomic levels. Screening prevalence was also lower in the highlands (80.9%), especially at an altitude exceeding 3,000 meters above sea level (83.1%). Additionally, individuals without health insurance (84.6%), those without a diagnosis of hypertension (77.3%), and daily smokers (83.1%) were less likely to be screened. Alcohol consumption in the last 30 days was not associated with screening prevalence (p = 0.7596).

Table 2. Factors associated with the lack of T2DM screening in the last 12 months: Bivariate analysis.

Variables	Glucose testing in the last 12 months		
	No (n = 19546)	Yes (n = 6 620)	p*
	n º (%) 74.7%	nº(%) 25.3%	
**Age**			
18 – 34 years	5 783 (79.8%)	1 468 (20.2%)	<0.001
35 or more years	13 769 (72.8%)	5 147 (27.2%)	
**Sex**			
Female	10 061 (73.3%)	3 666 (26.7%)	0.002
Male	9 490 (76.3%)	2 949 (23.7%)	
**Area of residence**			
Rural	4 140 (85.7%)	688 (14.3%)	<0.001
Urban	15 409 (72.2%)	5 927 (27.8%)	
**Education level**			
No education	843 (80.5%)	204 (19.5%)	<0.001
Incomplete elementary	3 297 (79.9%)	830 (20.1%)	
Complete elementary	1 287 (87.4%)	186 (12.6%)	
Incomplete secondary	2 601 (78.1%)	730 (21.9%)	
Complete secondary	5 730 (76.9%)	1 724 (23.1%)	
Higher education	5 793 (66.3%)	2 938 (33.7%)	
**Ethnicity**			
Native	5 694 (77.6%)	1 643 (22.4%)	<0.001
Zambo, afrodescendant	2 360 (78.7%)	639 (21.3%)	
White	1 376 (74.8%)	463 (25.2%)	
Mestizo	8 839 (71.6%)	3 512 (28.4%)	
Other	249 (75.9%)	79 (24.1%)	
Do not know/No response	1 034 (78.7%)	280 (21.3%)	
**Economic level**			
The poorest	4 210 (87.0%)	628 (13%)	<0.001
Poor	4 189 (80.3%)	1 028 (19.7%)	

Middle	4 228 (76.1%)	1 327 (23.9%)	
Rich	3 807 (70.2%)	1 614 (29.8%)	
The richest	3 116 (60.7%)	2 017 (39.3%)	
**Region of origin**			
Metropolitan Lima	6 863 (70.1%)	2 925 (29.9%)	<0.001
Rest of the coast	5 050 (73.8%)	1 798 (26.3%)	
Highlands	5 147 (80.9%)	1 217 (19.1%)	
Jungle	2 488 (78.6%)	678 (21.4%)	
**Altitude**			
Less than 3 000 m.a.s.l.	16 328 (73.2%)	5 963 (26.8%)	<0.001
Great or equal to 3 000 m.a.s.l.	3 224 (83.1%)	654 (16.9%)	
**Health insurance**			
Yes	15 637 (72.6%)	5 903 (27.4%)	<0.001
No	3 914 (84.6%)	712 (15.4%)	
**Hypertension**			
Present	1 622 (54.4%)	1 361 (45.6%)	<0.001
Absent	17 924 (77.3%)	5 259 (22.7%)	
**Daily smoker**			
No	319 (83.1%)	65 (16.9%)	0.0473
Si	19 232 (74.6%)	6 549 (25.4%)	
**Alcohol drinking**			
Present	7 439 (74.5%)	2 543 (25.5%)	0.7596
Absent	12 110 (74.8%)	4 071 (25.2%)	

*p-value from chi-square test

Multivariate analysis using Poisson regression models to calculate crude (cPR) and adjusted prevalence ratios (aPR) identified several factors associated with the lack of screening (Fig 2). These were: age between 18-34 years (aPR: 1.08; 95%CI: 1.05-1.11), male sex (aPR: 1.00063; 95%CI: 1.00063-1.5), belonging to the lowest wealth quintile (aPR: 1.09; 95%CI: 1.05-1.13), having only elementary education (aPR: 1.18; 95%CI: 1.13-1.23), living at an altitude greater than 3,000 m.a.s.l (aPR: 1.04; 95%CI: 1.004-1.07), not having a health insurance (aPR: 1.15; 95%CI: 1.12-1.18), and being a daily smoker (aPR: 1.10; 95%CI: 1.01-1.19). Factors that increased the prevalence of screening included having hypertension (aPR: 0.73; 95%CI: 0.68-1.78) and being in the two highest wealth quintiles (fourth quintile aPR: 0.93; 95%CI: 0.90-0.97; fifth quintile aPR: 0.85; 95%CI: 0.81-0.89). No significant association was found for ethnicity, area of residence, or region of origin. Further details can be found in Table 3.

Table 3. Factors associated with the non-screening for T2DM: Multivariate analysis.

Variables	Crude model			Adjusted model		
	PR	CI 95%	p	PR	CI 95%	p

**Age**						
35 or more years	Ref.			Ref.		
18 – 34 years	1.10	1.07 - 1.12	<0.001	1.08	1.05 - 1.12	<0.001
**Sex**						
Female	Ref.			Ref.		
Male	1.04	1.01 - 1.07	0.002	1.03	1.00 - 1.05[a]	0.044
**Area of residence**						
Urban	Ref.			Ref.		
Rural	1.19	1.16 - 1.21	<0.001	1.01	0.99 - 1.04	0.320
**Education level**						
Higher education	Ref.			Ref.		
No education	1.21	1.15 - 1.29	<0.001	1.16	1.09 - 1.23	<0.001
Incomplete elementary	1.20	1.16 - 1.25	<0.001	1.15	1.09 - 1.20	<0.001
Complete elementary	1.32	1.26 - 1.37	<0.001	1.18	1.13 - 1.23	<0.001
Incomplete secondary	1.18	1.12 - 1.24	<0.001	1.11	1.05 - 1.16	<0.001
Complete secondary	1.16	1.11 - 1.20	<0.001	1.09	1.05 - 1.13	<0.001
**Ethnicity**						
Mestizo	Ref.			Ref.		
Native	1.08	1.05 - 1.12	<0.001	0.99	0.96 - 1.03	0.658
Zambo, afro descendant	1.10	1.06 - 1.14	<0.001	1.02	0.98 - 1.05	0.388
White	1.05	0.99 - 1.14	0.105	1.01	0.94 - 1.05	0.769
Other	1.06	0.95 - 1.19	0.318	1.01	0.90 - 1.14	0.971
**Economic level**						
Middle	Ref.			Ref.		
The poorest	1.14	1.11 - 1.18	<0.001	1.09	1.05 - 1.13	<0.001
Poor	1.06	1.02 - 1.09	0.001	1.03	0.99 - 1.06	0.078
Rich	0.92	0.88 - 0.96	<0.001	0.93	0.90 - 0.97	0.001
The richest	0.80	0.76 - 0.84	<0.001	0.85	0.81 - 0.89	<0.001
**Region of origin**						
Metropolitan Lima	Ref.			Ref.		
Rest of the coast	1.05	1.02 - 1.09	0.006	0.98	0.95 - 1.02	0.328
Highlands	1.15	1.12 - 1.19	<0.001	1.00	0.97 - 1.05	0.709
Jungle	1.12	1.08 - 1.16	<0.001	0.98	0.95 - 1.02	0.320
**Altitude**						
Less than 3 000 m.a.s.l.	Ref.			Ref.		
Great or equal to 3 000 m.a.s.l.	1.14	1.12 - 1.16	<0.001	1.04	1.00 - 1.07[b]	0.028
**Health insurance**						
Yes	Ref.			Ref.		
No	1.17	1.13 - 1.20	<0.001	1.15	1.12 - 1.18	<0.001
**Hypertension**						
Absent	Ref.			Ref.		
Present	0.71	0.66 - 0.75	<0.001	0.73	0.68 - 0.78	<0.001
**Daily smoker**						
No	Ref.			Ref.		
Si	1.11	1.02 - 1.21	0.017	1.10	1.01 - 1.19	0.029

PR = Prevalence Ratio, Ref. = Reference category

Prevalence Ratios are calculated with non-screening as the main dependent outcome

[a]Male Sex: IC95% (1.00063 - 1.051108)

[b]Altitude “Greater or equal to 3000 m.a.s.l.: IC95% (1.003953 - 1.072157)

Fig 2. Forest Plot for Factors associated with the lack of screening for T2DM

Discussion

Our analysis of a representative sample of the Peruvian population found that the T2DM screening prevalence was only 25.3%. This represents a lower prevalence compared to similar studies in the nation. In an analysis of the Peruvian National Health survey in 2020, Trujillo-Minaya reported a T2DM screening prevalence of 27.7% [10]. However, this study did not apply any specific T2DM screening criteria and included all individuals over 15 years old, likely including a broader population potentially explaining the higher prevalence. Similarly, Aedo found a screening prevalence of 30.6% among individuals over 45 years old with data from ENDES 2014 [11]. The differences in age cutoffs and screening criteria can partially explain the variations in prevalence. To our knowledge, our study is the first one that considers the updated ADA T2DM screening recommendations in Peru, which are the most sensitive screening guidelines and therefore facilitate greater detection of disease. The insufficient coverage of preventive medicine services in Peru, which includes an unequal and insufficient distribution of medical doctors in primary care, is an ongoing healthcare barrier that could contribute to this decline in screening, specifically by decreasing access to preventive services in non-centric areas of the nation [12]. Additionally, the impact of the COVID-19 pandemic on outpatient services in the nation was substantial, with many hospitals limiting outpatient visits, which could have limited preventive visits and thus screening rates, with an effect expanding to the subsequent post-pandemic years [13]. This low screening prevalence signifies a public health challenge, as the prevalence of T2DM has been increasing in the nation.

To provide further context, diabetes screening prevalence has also been studied in other Latin American countries, though methodological differences make comparisons difficult. In Mexico, Villalobos reported that 15.2% of adults over 20 years old attended preventive healthcare services modules for the detection of T2DM with data from the National Health and Nutrition Survey of 2018-2019 [14]. In Brazil, Santos analyzed a National Health Survey and documented that more than 70% of adults underwent a glucose test in the two years prior to being surveyed [15]. This difference in screening prevalence may be due to different ways of conceptualizing T2DM screening, question formulation and timeframe of screening (screening in 1 year vs in the last 2 years). However, the

difference in reported prevalence is considerable and the different healthcare systems, preventive coverages and emphasis on primary care in each nation could contribute to this varying prevalence. For instance, the unified health system in Brazil provides universal access to all levels of care with increasing coverage throughout the years, facilitating ample access to medical consultation and thus screening [15].

Regarding the factors associated with the lack of T2DM screening, we found that younger individuals (ages 18 to 34) were less likely to undergo screening. This finding has been described before, with studies describing higher attendance to medical consultations and annual preventive check-ups among older populations, both in men and women [10,14,16]. This could be attributed to the perception that younger people are healthier or their tendency not to seek medical attention unless they experience pain or symptoms that limit their daily activities, with limited emphasis on preventive consultation [17–21]. This finding is concerning, as the incidence of diabetes in individuals aged 40 or less has been increasing in recent years, which highlights the importance of increasing screening prevalence in younger populations to identify the disease early in its course [22].

Our study found that males had a 3% higher prevalence of not undergoing T2DM screening. This finding aligns with studies from Peru, Canada and Brazil, in which men were less likely to undergo T2DM screening compared to women [10,16,21,23]. Some barriers to preventive care in men in middle income countries are a lack of concern for health, limited financial resources, and poor initiative from health personnel in promoting screening, especially among young men [18]. These barriers are also shared by some of the Latino male population, with higher emphasis in the rural setting [24]. In Brazil, Cobo reported that many men claim that their jobs do not align with the health centers schedules, limiting their medical consultations as they have to prioritize economically productive activities [21]. Finally, cultural and social perceptions such as the “machista” mentality which associates illness with weakness could be another barrier preventing adequate screening in men, in which obtaining preventive care and screening could be perceived as incompatible with traditional male roles [20,24].

Educational level and socioeconomic status are among the most widely associated factors with the lack of screening for various pathologies. Studies in Peru have reported that T2DM screening was more likely as the educational level and socioeconomic status of the population increased, which aligns

to our findings [10,11]. In our study, the prevalence of unscreened individuals was highest among those with only elementary education (aPR: 1.18) and those with no formal education (aPR: 1.16) when compared to individuals with higher education. The difference in screening rates between educational levels may be due to increased health literacy among those with higher education, which can increase preventive visits and chance for receiving screening [17,19]. Additionally, those with lower education and socioeconomic level may perceive screening as unnecessary or financially burdensome, contributing to lower screening rates [17]. Concerning socioeconomic status, individuals in the lowest wealth quintile had the highest prevalence ratio for being unscreened. These results are consistent with findings from studies in Asian populations, where lower income was associated with a lower likelihood of attending T2DM screening among young adults [17]. Lower socioeconomic status decreases the likelihood of screening possibly due to limited healthcare access and greater financial constraints regarding transportation costs and lost income when attending a medical visit in this population [14,16,19].

The lack of health insurance has also been associated with lower screening for T2DM and other non-communicable diseases. A lower prevalence of health service use and medical visits among individuals without health insurance has been described, regardless of any prior diagnosis of a non-communicable disease [25]. In Peru, efforts have been made to increase equitable access to healthcare, with an increase in the healthcare insurance coverage in the last decade, reaching approximately 76% of the population covered by any type of health insurance in 2017 [26]. Despite this coverage, the use of medical consultation and participation in preventive medicine is still low, reflecting the persistent barriers to access despite having health insurance [27]. While it is important to increase health insurance coverage, it is mandatory to address the barriers to healthcare access such as long waiting times, quality of care and centralization of healthcare.

Among other factors, we found daily smokers had a 10% higher prevalence of not undergoing T2DM screening compared to non-smokers. Medical literature indicates that unhealthy habits, such as excessive alcohol consumption or tobacco use are predictive factors for lack of medical consultation and screening [14,19,28]. T2DM screening efforts should be emphasized in this population despite not being considered a formal screening criterion. Additionally, the prevalence of unscreened individuals

was lower among those previously diagnosed with hypertension. This matches with what was reported by medical literature [14,19,25]. One explanation could be the greater opportunities for education on the importance of regular preventive check-ups, due to the higher number of medical consultations that individuals with chronic illnesses have, and because the comorbidity between hypertension and diabetes prompts for screening of the latter [15,19,25].

An interesting finding in our study is the lack of a significant association between the area of residence (urban vs. rural) and T2DM screening. However, we found a 4% higher prevalence of unscreened individuals among populations residing at altitudes of 3,000 m.a.s.l or higher. While global literature supports the existence of an association between living in a rural area and a lack of routine medical check-ups, in our context, the altitude of residence may be a more determining barrier due to the difficult access (related to distance and high transportation costs) to medical services experienced in many Andean communities, regardless of whether they are classified as rural or urban areas. Additionally, cultural and health-seeking behaviors differ in the high-altitude Andean communities, which exhibit a higher reliance on traditional medicine and engage less in health promotion strategies for non-communicable diseases [29]. The strengthening and expansion of primary care centers, mobile health units and telemedicine services can serve as strategies to mitigate the impact of these factors and increase T2DM screening.

This study has several strengths. First, this study benefits from a large nationally representative sample obtained through a well-structured probabilistic sampling method. Second, to our knowledge, it is the first study to determine the T2DM screening prevalence in Peru according to the latest screening criteria proposed by the ADA, providing an updated assessment of screening coverage in the nation. Finally, it is one of the few population level studies that explores various health determinants in the prevention of noncommunicable diseases in Peru, which contributes valuable insights for public health planning.

This study is not exempt from limitations. First, as a cross-sectional study, it cannot establish a temporal sequence in the associations found. As in any questionnaire, recall bias can also lead to under-reporting of the event. Additionally, the definition of the outcome is based on self-reported glucose testing in the last year, which does not directly reflect the screening for this disease: a person may have

had a glucose test two or three years ago, and still meet ADA recommendations (glucose should be measured every three years if the first screening was negative), or had their glucose measured in a context outside of diabetes screening (for example, when hospitalized for another reason). Nevertheless, we consider it the closest approximation to T2DM screening with the information available in the health survey. Finally, the ENDES does not inquire for other factors that could be associated with T2DM screening, such as a family history of T2DM, which has been shown to increase the screening rate among individuals.

Conclusion

Only 25.3% of the population that met the ADA diabetes screening criteria, underwent screening in Peru in 2022. The factors associated with the lack of screening included being in the age range of 18 to 34 years, being male, having a low level of education, belonging to the lower wealth quintiles, lacking health insurance, living at an altitude of 3,000 m.a.s.l or higher, and being a daily smoker. In this regard, efforts should be made to promote public policies that raise awareness about the importance of early diagnosis of T2DM and that target the population with the aforementioned characteristics. Public health strategies could include updating national diabetes screening recommendations to align with international guidelines, expansion of primary health coverage, implementation of community-based screening campaigns and strengthening health education promotion. This would facilitate early detection and treatment of disease and ultimately decrease the burden of T2DM in Peru.

Acknowledgements

The authors do not cite any acknowledgements.

References

1. Herman WH, Ye W, Griffin SJ, Simmons RK, Davies MJ, Khunti K, Rutten GEHM, Sandbaek A, Lauritzen T, Borch-Johnsen K, Brown MB, Wareham NJ. Early Detection and Treatment of

Type 2 Diabetes Reduce Cardiovascular Morbidity and Mortality: A Simulation of the Results of the Anglo-Danish-Dutch Study of Intensive Treatment in People With Screen-Detected Diabetes in Primary Care (ADDITION-Europe). Diabetes Care. 2015 May 15;38(8):1449–55.

2. Nanayakkara N, Curtis AJ, Heritier S, Gadowski AM, Pavkov ME, Kenealy T, Owens DR, Thomas RL, Song S, Wong J, Chan JCN, Luk AOY, Penno G, Ji L, Mohan V, Amutha A, Romero-Aroca P, Gasevic D, Magliano DJ, Teede HJ, Chalmers J, Zoungas S. Impact of age at type 2 diabetes mellitus diagnosis on mortality and vascular complications: systematic review and meta-analyses. Diabetologia. 2021 Feb;64(2):275–87.
3. American Diabetes Association Professional Practice Committee. 2. Diagnosis and Classification of Diabetes: Standards of Care in Diabetes—2024. Diabetes Care. 2023 Dec 11;47(Supplement_1):S20–42.
4. Rios-Blancas MJ, Pando-Robles V, Razo C, Carcamo CP, Mendoza W, Pacheco-Barrios K, Miranda JJ, Lansingh VC, Demie TG, Saha M, Okonji OC, Yigit A, Cahuana-Hurtado L, Chacón-Uscamaita PR, Bernabe E, Culquichicon C, Chirinos-Caceres JL, Cárdenas R, Alcalde-Rabanal JE, Barrera FJ, Quintanilla BPA, Shorofi SA, Wickramasinghe ND, Ferreira N, Almidani L, Gupta VK, Karimi H, Alayu DS, Benziger CP, Fukumoto T, Mostafavi E, Redwan EMM, Gebrehiwot M, Khatab K, Koyanagi A, Krapp F, Lee S, Noori M, Qattea I, Rosenthal VD, Sakshaug JW, Wagaye B, Zare I, Ortega-Altamirano DV, Murillo-Zamora E, Vervoort D, Silva DAS, Oulhaj A, Herrera-Serna BY, Mehra R, Amir-Behghadami M, Adib N, Cortés S, Dang AK, Nguyen BT, Mokdad AH, Hay SI, Murray CJL, Lozano R, García PJ. Estimating mortality and disability in Peru before the COVID-19 pandemic: a systematic analysis from the Global Burden of the Disease Study 2019. Front Public Health. 2023 Jun 22;11:1189861.
5. Seclen SN, Rosas ME, Arias AJ, Huayta E, Medina CA. Prevalence of diabetes and impaired fasting glucose in Peru: report from PERUDIAB, a national urban population-based longitudinal study. BMJ Open Diabetes Res Care. 2015 Oct 1;3(1):e000110.
6. Instituto Nacional de Estadistica e Informatica. Peru: Enfermedades No Transmisibes y Transmisibles 2022 [Internet]. INEI; 2023 [cited 2024 Nov 4]. Available from: https://www.inei.gob.pe/media/MenuRecursivo/publicaciones_digitales/Est/Lib1951/libro.pdf
7. Ministerio de Salud del Peru. GUÍA DE PRÁCTICA CLÍNICA PARA EL DIAGNÓSTICO, TRATAMIENTO Y CONTROL DE LA DIABETES MELLITUS TIPO 2 EN EL PRIMER NIVEL DE ATENCIÓN [Internet]. 2016 [cited 2024 Nov 4]. Available from: https://bvs.minsa.gob.pe/local/MINSA/3466.pdf
8. Villena JE. Diabetes Mellitus in Peru. Ann Glob Health. 2015 Nov 1;81(6):765–75.
9. Gnardellis C, Notara V, Papadakaki M, Gialamas V, Chliaoutakis J. Overestimation of Relative Risk and Prevalence Ratio: Misuse of Logistic Modeling. Diagnostics. 2022 Nov 17;12(11):2851.
10. Trujillo-Minaya F, Vera-Ponce VJ, Torres-Malca JR, Zuzunaga-Montoya FE, Valencia JG, Cruz-Vargas JADL, Cruz-Ausejo L. Factores asociados al cribado de Diabetes Mellitus en población Peruana ¿problema para la salud pública? Rev Cuid [Internet]. 2023 Mar 15 [cited 2024 Nov 4];14(1). Available from: https://revistas.udes.edu.co/cuidarte/article/view/2792
11. Aedo Tito KP, Conde Bazán LF. Inequidades en el tamizaje para Diabetes Mellitus: estudio transversal de base poblacional en Perú, 2014. Repos Académico - UPC [Internet]. 2018 Jan 18 [cited 2024 Nov 4]; Available from: https://repositorioacademico.upc.edu.pe/handle/10757/622888
12. Oyola García AE. Desigualdad en la distribución de médicos en el Perú. Rev Cuba Salud Pública [Internet]. 2021 Mar [cited 2025 Feb 10];47(1). Available from: http://scielo.sld.cu/scielo.php?script=sci_abstract&pid=S0864-34662021000100003&lng=es&nrm=iso&tlng=es
13. Lopez-Huamanrayme E, Salsavilca-Macavilca E, Taype-Rondan A, Lopez-Huamanrayme E, Salsavilca-Macavilca E, Taype-Rondan A. Impacto de la pandemia por COVID-19 en la consulta ambulatoria endocrinológica y teleconsulta en un hospital Peruano. Rev Cuerpo Méd Hosp Nac Almanzor Aguinaga Asenjo. 2022 Jul;15(3):349–55.
14. Villalobos A, Aguilar-Salinas CA, Martínez MR, Rojas-Martínez R. Perfiles poblacionales asociados con la asistencia a servicios preventivos para tamizaje de diabetes e hipertensión. Ensanut 2018-19. Salud Pública México. 2020 Nov 24;62(6, Nov-Dic):754–66.
15. Santos KBM, Reis RCP dos, Duncan BB, D’Avila OP, Schmidt MI. Access to diabetes diagnosis

in Brazil based on recent testing and consultation: The Brazilian national health survey, 2013 and 2019. Front Endocrinol [Internet]. 2023 Mar 22 [cited 2024 Nov 4];14. Available from: https://www.frontiersin.org/journals/endocrinology/articles/10.3389/fendo.2023.1122164/full

16. Dryden R, Williams B, McCowan C, Themessl-Huber M. What do we know about who does and does not attend general health checks? Findings from a narrative scoping review. BMC Public Health. 2012 Aug 31;12(1):723.
17. AshaRani PV, Devi F, Wang P, Abdin E, Zhang Y, Roystonn K, Jeyagurunathan A, Subramaniam M. Factors influencing uptake of diabetes health screening: a mixed methods study in Asian population. BMC Public Health. 2022 Aug 9;22(1):1511.
18. Teo CH, Ng CJ, White A. Factors influencing young men's decision to undergo health screening in Malaysia: a qualitative study. BMJ Open. 2017 Mar 10;7(3):e014364.
19. Paek SC, Zhang NJ. Factors associated with the use of annual health checkups in Thailand: evidence from a national cross-sectional health and welfare survey. Front Public Health [Internet]. 2024 May 10 [cited 2024 Nov 4];12. Available from: https://www.frontiersin.org/journals/public-health/articles/10.3389/fpubh.2024.1390125/full
20. Harvey IS, Alston RJ. Understanding preventive behaviors among mid-Western African-American men: a pilot qualitative study of prostate screening. J Mens Health. 2011 May 1;8(2):140.
21. Cobo B, Cruz C, Dick PC. Gender and racial inequalities in the access to and the use of Brazilian health services. Cienc Saude Coletiva. 2021 Sep;26(9):4021–32.
22. Strati M, Moustaki M, Psaltopoulou T, Vryonidou A, Paschou SA. Early onset type 2 diabetes mellitus: an update. Endocrine. 2024;85(3):965–78.
23. Kaul P, Chu LM, Dover DC, Yeung RO, Eurich DT, Butalia S. Disparities in adherence to diabetes screening guidelines among males and females in a universal care setting: A population-based study of 1,380,697 adults. Lancet Reg Health Am. 2022 Oct;14:100320.
24. Daniel-Ulloa J, Reyes JA, Morales-Campos DY, Villareal E, López Cevallos DF, Hernandez H, Baquero B. Rural Latino Men's Experiences and Attitudes Toward Health: A Pilot Photovoice Study. Am J Mens Health. 2023 Mar 9;17(2):15579883231158525.
25. Malta DC, Bernal RTI, Lima MG, Araújo SSC de, Silva MMA da, Freitas MI de F, Barros MB de A. Noncommunicable diseases and the use of health services: analysis of the National Health Survey in Brazil. Rev Saúde Pública. 2017 Jun 1;51:4s.
26. Mezones-Holguín E, Amaya E, Bellido-Boza L, Mougenot B, Murillo JP, Villegas-Ortega J, Sara JCDC. [Health insurance coverage: the peruvian case since the universal insurance act]. Rev Peru Med Exp Salud Publica. 2019;36(2):196–206.
27. Díaz-Ruiz R, Vargas-Fernández R, Rojas-Roque C, Hernández-Vásquez A. Socioeconomic inequalities in the use of medical consultation services in Peru, 2019. Int J Equity Health. 2024 Jan 20;23(1):10.
28. Thorogood M, Coulter A, Jones L, Yudkin P, Muir J, Mant D. Factors affecting response to an invitation to attend for a health check. J Epidemiol Community Health. 1993 Jun;47(3):224–8.
29. Sanchez-Samaniego G, Hartinger SM, Mäusezahl D, Hattendorf J, Fink G, Probst-Hensch N. Prevalence, awareness, treatment and control of high blood pressure in a cohort in Northern Andean Peru. Glob Health Action. 2023 Dec 1;16(1):2285100.

Supporting information

S1 Data. Raw databases for Household information

S2 Data. Raw databases for Health information

S1 Table. Definition and Operationalization of Variables

Fig 1. Flowchart of participant selection

Click here to access/download;Figure;Fig1.tiff

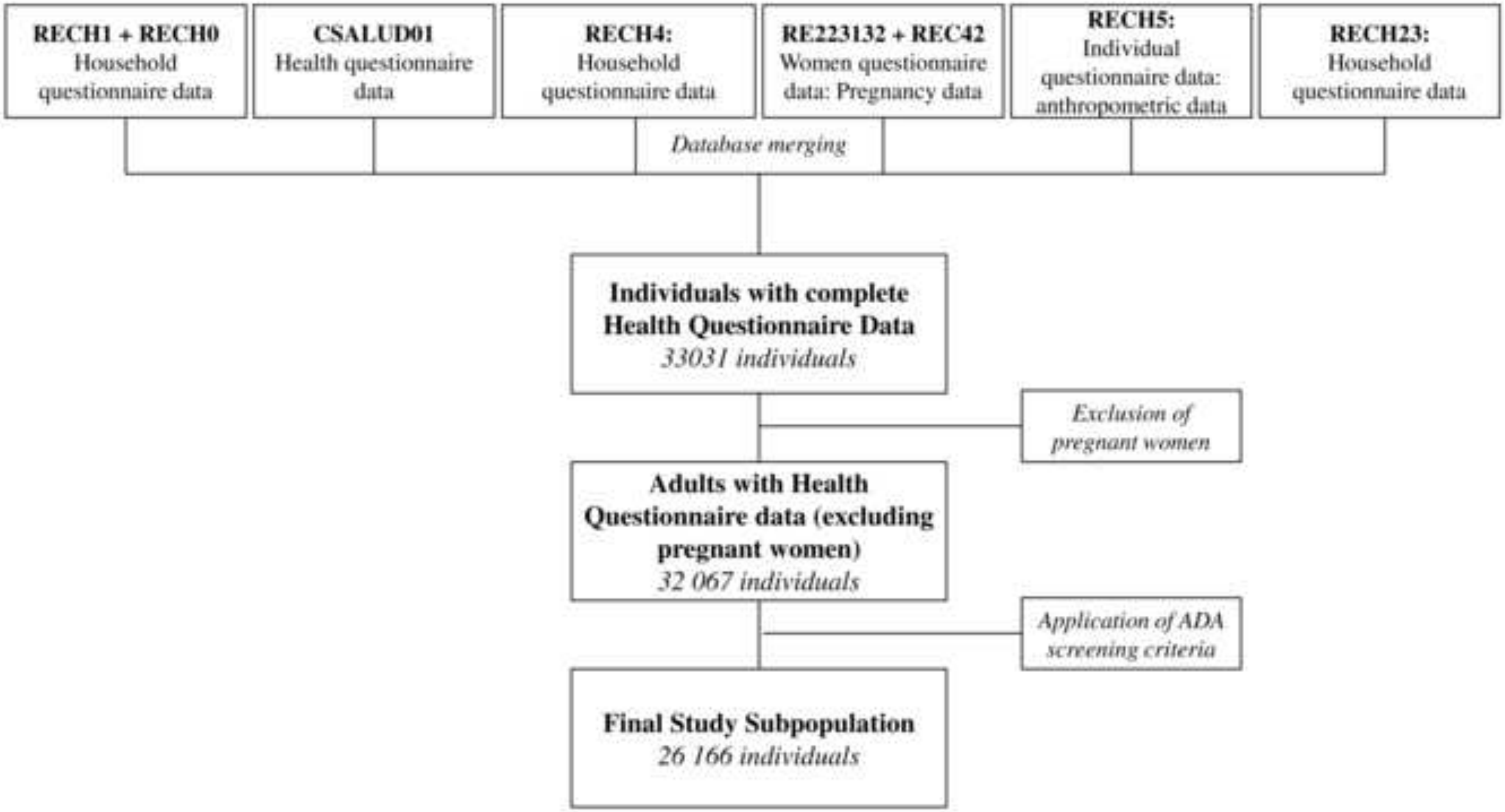

Fig 2. Forest Plot for Factors associated with the lack of screening for T2DM

Click here to access/download;Figure;Fig2.tif

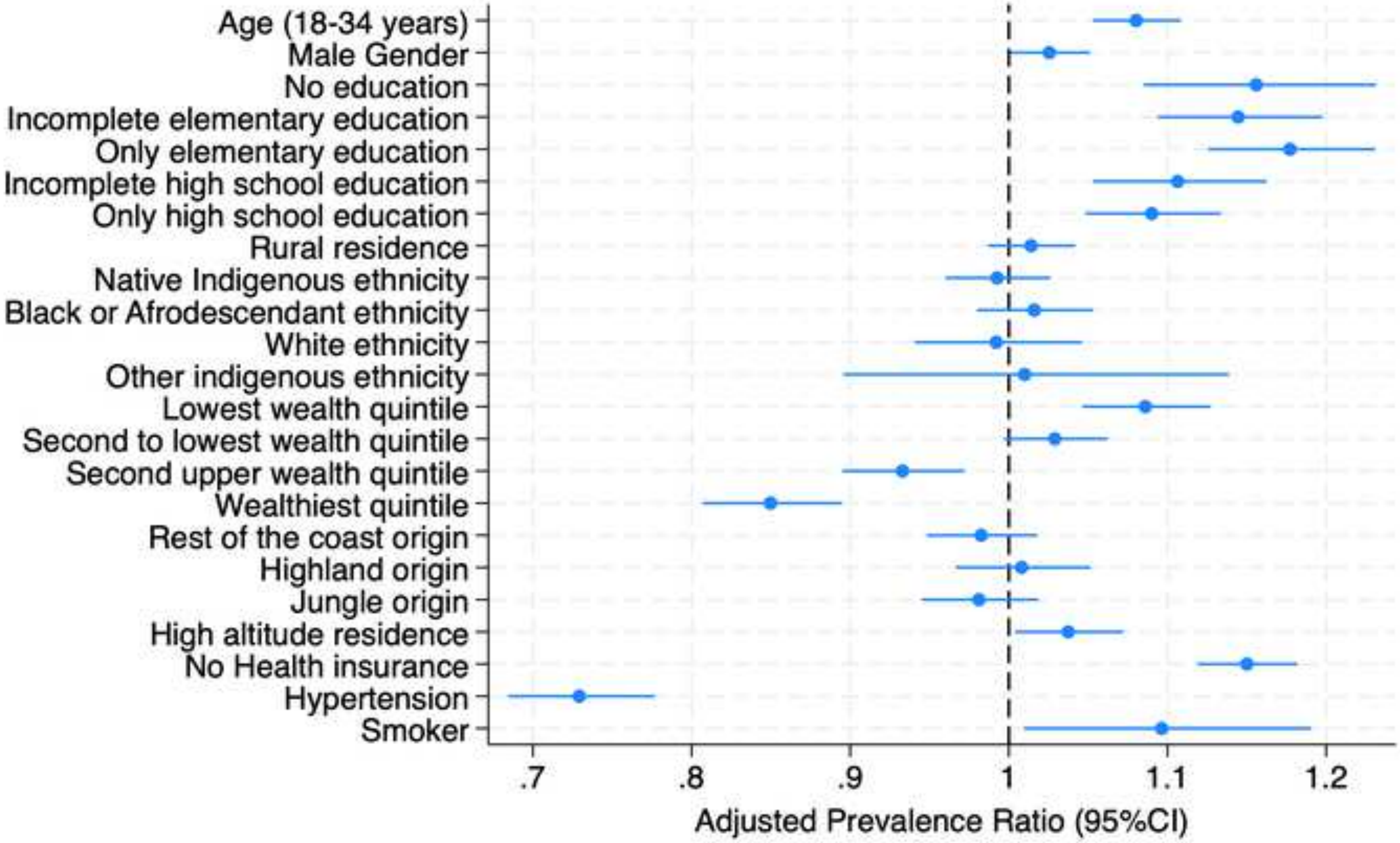

S1 Table. Definition and Operationalization of Variables

Click here to access/download
Supporting Information
S1_Table.docx

S1 Data. Raw databases for Household information

Click here to access/download

Supporting Information - Compressed/ZIP File Archive

S1_Data.zip

S2 Data. Raw databases for Health information

Click here to access/download

Supporting Information - Compressed/ZIP File Archive

S2_Data.zip
