# Supplementary material for: Diabetes Mellitus screening and associated factors in Peru: A cross-sectional analysis of a national health survey
Source: PLOS Glob Public Health. 2025 Sep 3;5(9):e0005150. doi: 10.1371/journal.pgph.0005150 (PMC12407462; doi:10.1371/journal.pgph.0005150)
Supplement: Attachment — Submitted filename: PGPH-D-25-00415_R2rev.pdf [file pgph.0005150.s008.pdf]

PLOS Global Public Health

Diabetes Mellitus screening and associated factors in Peru: a cross-sectional analysis of a national health survey

--Manuscript Draft--

**Manuscript Number:**	PGPH-D-25-00415R2
**Article Type:**	Research Article
**Full Title:**	Diabetes Mellitus screening and associated factors in Peru: a cross-sectional analysis of a national health survey
**Short Title:**	Diabetes Mellitus screening in Peru: cross-sectional analysis of a national health survey
**Corresponding Author:**	Einer Carlos Eduardo Arevalo Rios, M.D.<br>Universidad Peruana Cayetano Heredia Facultad de Medicina<br>Lima, PERU
**Order of Authors:**	Einer Carlos Eduardo Arevalo-Rios, M.D.
	Jennifer Layza-Reyes, M.D.
	Victor Hugo Noriega-Ruiz, M.D.
**Keywords:**	Diabetes Mellitus; screening; Peru
**Abstract:**	Screening rates for Type 2 diabetes mellitus (T2DM) in Peru remain low despite an increasing prevalence of the disease. In 2022, the American Diabetes Association (ADA) recommended screening adults aged 18-34 with overweight or obesity and at least one risk factor, including Latino ethnicity and lowered the universal screening age to 35 years. This study aimed to determine the T2DM screening prevalence in Peru and the factors associated with lack of screening in the population that meets ADA screening criteria. A cross-sectional, secondary data analysis was conducted based on the 2022 Peruvian Demographic and Health Survey database. Screening was defined as apparently healthy individuals having their blood glucose measured in the last year. Individuals aged 18-34 years overweight or obese and all individuals aged ≥ 35 years were included. Bivariate analysis was performed using the chi-squared test, and multivariate Poisson regression was used to estimate prevalence ratios (PRs), adjusting for potential confounders. Of 26,166 individuals who met inclusion criteria, 25.3% were screened for T2DM. The factors most strongly associated with lack of screening were: age 18-34 years (aPR: 1.08; 95% CI: 1.05-1.11), having only elementary education (aPR: 1.18; 95% CI: 1.13-1.23), not having health insurance (aPR: 1.15; 95% CI: 1.12-1.18), belonging to the lowest wealth quintile (aPR: 1.09; 95% CI: 1.05-1.13), and daily smoking (aPR: 1.10; 95% CI: 1.01-1.19). The national rate of screening for T2DM in Peru is low (25.3%). There is a higher prevalence for a lack of screening in people who: are younger, have only primary education, do not have health insurance, belong to the lowest wealth quintile, and are smokers. Targeted interventions are needed to improve screening coverage in these high-risk populations.
**Additional Information:**	
**Question**	**Response**
**Financial Disclosure**<br><br>Enter a financial disclosure statement that describes the sources of funding for the work included in this submission and the role the funder(s) played. This includes grants and any commercial funding of the work or authors.	The author(s) received no specific funding for this work.

Powered by Editorial Manager® and ProduXion Manager® from Aries Systems Corporation

This statement will be typeset if the manuscript is accepted for publication.<br><br>*Please review the submission guidelines and the instructions link below for detailed requirements and guidance.*	
**Competing Interests**<br><br>On behalf of all authors, disclose any competing interests that could be perceived to bias this work.<br><br>This statement will be typeset if the manuscript is accepted for publication.<br><br>*Please review the instructions link below and PLOS Global Public Health's competing interests policy to determine what information must be disclosed at submission.*	The authors have declared that no competing interests exist.
**Data Availability**<br><br>Before publication, Authors are required to make fully available and without restriction all data underlying their findings. Please see our PLOS Data Policy page for detailed information on this policy.<br><br>A **Data Availability Statement**, detailing where the data can be accessed, is required at first submission. Insert your Data Availability Statement in the box below.<br><br>Please see the data reporting section of our submission guidelines for instructions on what you need to include in your Data Availability Statement.<br><br>This statement will be typeset if the	We are submitting the raw data from our analysis in CSV format; these files are included as supporting information.

manuscript is accepted for publication.<br><br>*PLOS allows rare exemptions to address legal and ethical concerns. If you have legal or ethical restrictions, please use the box below to detail these in full sentences for the Journal team to consider.*	

Powered by Editorial Manager® and ProduXion Manager® from Aries Systems Corporation

Diabetes Mellitus screening and associated factors in Peru: a cross-sectional analysis of a national health survey

Einer Carlos Eduardo Arevalo-Rios[1*], Jennifer Layza-Reyes [1], Victor Hugo Noriega-Ruiz [1,2]

[1] Alberto Hurtado School of Medicine, Universidad Peruana Cayetano Heredia, Lima, Peru

[2] Department of Endocrinology, Clinica Anglo Americana, Lima, Peru

* Corresponding author

E-mail: einer.arevalo@upch.pe (EA)

Abstract

Screening rates for Type 2 diabetes mellitus (T2DM) in Peru remain low despite an increasing prevalence of the disease. In 2022, the American Diabetes Association (ADA) recommended screening adults aged 18-34 with overweight or obesity and at least one risk factor, including Latino ethnicity and lowered the universal screening age to 35 years. This study aimed to determine the T2DM screening prevalence in Peru and the factors associated with lack of screening in the population that meets ADA screening criteria. A cross-sectional, secondary data analysis was conducted based on the 2022 Peruvian Demographic and Health Survey database. Screening was defined as apparently healthy individuals having their blood glucose measured in the last year. Individuals aged 18-34 years overweight or obese and all individuals aged ≥ 35 years were included. Bivariate analysis was performed using the chi-squared test, and multivariate Poisson regression was used to estimate prevalence ratios (PRs), adjusting for potential confounders. Of 26,166 individuals who met inclusion criteria, 25.3% were screened for T2DM. The factors most strongly associated with lack of screening were: age 18-34 years (aPR: 1.08; 95% CI: 1.05-1.11), having only elementary education (aPR: 1.18; 95% CI: 1.13-1.23), not having health insurance (aPR: 1.15; 95% CI: 1.12-1.18), belonging to the lowest wealth quintile (aPR: 1.09; 95% CI: 1.05-1.13), and daily smoking (aPR: 1.10; 95% CI: 1.01-1.19). The national rate of screening for T2DM in Peru is low (25.3%). There is a higher prevalence for a lack of screening in people who: are younger, have only primary education, do not have health insurance, belong to the lowest wealth quintile, and are smokers. Targeted interventions are needed to improve screening coverage in these high-risk populations.

Introduction

Diabetes Mellitus is one of the leading causes of mortality worldwide. However, early diagnosis and treatment can significantly reduce morbidity and mortality [1]. Longer periods without adequate metabolic control increase the risk of developing chronic complications that worsen prognosis [1,2]. Screening with a fasting plasma glucose measurement, glycated hemoglobin (HbA1c), or glucose

tolerance test is the main strategy for early detection of this disease. In 2022, the ADA modified its screening recommendations to include universal screening for all individuals starting at age 35; and targeted screening for individuals over 18 years old with overweight or obesity and at least one additional risk factor, including Latino ethnicity, which is considered high-risk [3]. These modifications were made to improve sensitivity of screening and to promote early detection of disease. Following these recommendations, and considering Peruvians as Latinos, all individuals with overweight or obesity between the ages of 18 and 34, as well as everyone over 35, should be screened for T2DM in Peru.

Peru is considered an upper-middle-income country with a geographically and ethnically diverse population that has persistent barriers to healthcare access despite recent economic growth [4]. Its diverse topography is marked by differences in the three main natural regions of the country: coast, highlands and jungle. Approximately 58% of the population resides on the coast, with many of the healthcare facilities concentrated in the capital districts of each department. The highlands of Peru are inhabited by 28.1% of the population, with many of them living at high altitudes, with limited infrastructures, deficient road networks and poor access to health services. The jungle of Peru, comprising 13% of the population, has many scattered native-indigenous communities with limited interconnection and many of them only accessible by air or water [4]. These characteristics pose an intrinsic barrier to healthcare access throughout the nation that impact early disease detection, including T2DM screening. Regarding non-communicable diseases, the prevalence of T2DM in Peru has increased in the past decades, with latest reports estimating a prevalence of 5-7 % highlighting variations among regions in the nation (coast, highlands, and jungle) [5,6]. It is hypothesized that socioeconomic and cultural barriers affect the T2DM screening coverage. It is currently unknown how many people have been screened in the country following the latest ADA guideline recommendations. While the Ministry of Health in Peru has defined screening criteria in national guidelines, these have not been updated since 2016, despite the increasing prevalence of the disease in the country [5,7]. Since T2DM is a disease that leads to complications that can be prevented with timely diagnosis and treatment, this study uses the latest and more sensitive screening criteria, which are the ADA screening recommendations. Therefore, in this study data from a nationally representative health survey was

analyzed to answer the following questions: What is the prevalence of diabetes screening in Peru, and what factors are associated with the lack of screening?

Materials and Methods

Study design and area

A cross-sectional analysis was performed using the 2022 Peruvian Demographic and Health Survey (DHS) database. This survey was conducted by the National Institute of Statistics and Informatics (INEI) from January to December 2022 on a nationally representative sample in all regions and departments of Peru. The DHS collected information through face-to-face interviews, and anthropometric measurements were obtained by trained individuals. The survey comprises multiple questionnaires; for this study, the Health Survey component was analyzed, as it includes data on non-communicable diseases such as T2DM. These datasets are publicly accessible at https://proyectos.inei.gob.pe/microdatos/ (S1-S2 Data).

Study population

The target population of the DHS are the usual residents of the interviewed households, which provide representative estimates of the population of Peru. In this study, complete-case responses from those who met the 2022 ADA screening criteria were analyzed, considering Latino/Hispanic ethnicity as a risk factor. Body Mass Indexes were calculated based on the anthropometric measures obtained in the interviews and as recorded in the Health Survey.

Inclusion and Exclusion criteria

Participants were included based on the two key populations to screen according to the ADA:

- Adults aged 18-34 years with overweight or obesity and one additional risk factor, specifically Latino ethnicity, who had complete data in the Health Survey.

- All individuals aged 35 years and older with complete data in the Health Survey.

Pregnant women, individuals with a previous diagnosis of T2DM and those taking antidiabetic medications were excluded.

Data collection and sample size

The DHS used a two-stage, probabilistic, balanced, stratified, and independent sampling design, covering all regions of Peru. The Health Survey was administered to 36 650 households and interviewed 31 917 individuals aged 15 years and over. This study involved no direct contact with participants, as it was based on the analysis of publicly available datasets. Only complete-case responses were analyzed.

Variables

The dependent variable of this study was the self-report of T2DM screening; based on the question: "In the past 12 months, has a doctor or other health professional measured your blood glucose or "sugar" level?". This outcome was categorized as a dichotomous variable.

Potential risk factors for the lack of screening were identified based on review of previous literature. These included age, sex, residence area, region of origin, altitude of residence, education level, ethnicity, wealth index and health insurance status. The wealth index was defined in quintiles as categorized in the DHS and based on a cumulative measure of a household's living standard. Age was further categorized in 18-34 and 35 years or older to reflect the distinct screening group recommendations of the ADA. Altitude of residence was categorized into living at an altitude more than 3 000 meters above sea level (m.a.s.l.) or not, based on previous studies that have reported lower diabetes prevalence at higher altitude in Peru [8]. Clinical variables affecting screening rates were included: previous hypertension diagnosis, current alcohol consumption, and smoking status. Further description of variables included in the survey is included as supplementary information (S1 Table).

Statistical analysis

Data was analyzed using STATA SE (18.0 version, College Station, Texas 77845, USA). To account for the complex sampling design of the DHS and the analysis of a specific subpopulation, the *svy* and *subpop* commands were used. Raw databases were merged to include the sociodemographic, anthropometric and health questionnaire information of surveyed individuals. Categorical variables are reported as frequencies and proportions, while quantitative variables are reported as mean and standard error for normally distributed data. Chi-square tests were used for the bivariate analysis between the independent variables and the dependent variable. Multivariate analysis was conducted using generalized linear models with Robust Poisson regression variance estimation to calculate crude and adjusted prevalence ratios for non-screening, considering the expected outcome prevalence to be greater than 10% [9]. The variables included in the multivariate model were the ones that showed association in the bivariate model and those epidemiologically relevant according to the literature review. A p-value ≤ 0.05 was considered statistically significant.

Ethical considerations

Data from the DHS is available publicly online as published by the INEI. When conducting the field interviews, the interviewers obtained written and informed consent from each respondent before participating in the survey. For this study, data was accessed on June 26th, 2024. The information collected did not allow for the identification of individuals. As this is a secondary study and no direct contact with participants was made, informed consent was not required. The protocol of this study was reviewed and approved by the Institutional Committee on Ethics in Human Research of the Peruvian University Cayetano Heredia in Lima, Peru.

Results

Characteristics of the study population

The subpopulation that met the ADA screening criteria (35 years or older and 18-34 years with overweight or obesity) was 26,166 individuals (Fig 1). Among them, the majority were women (52.5%), over 35 years old, resided in urban areas, identified as “Mestizo” ethnicity, and had health insurance. Approximately one-third had achieved higher education. The distribution of the socioeconomic status categories was homogeneous, while most lived in Metropolitan Lima. The majority did not smoke daily, 11.4% had been diagnosed with hypertension, and approximately one-third of individuals reported recent alcohol consumption in the past 30 days. The full description of the characteristics is shown in Table 1.

Fig 1. Flowchart of participant selection

Table 1. Characteristics of the population that met the ADA screening criteria

**Characteristics**	**Total** (N= 26 166)	**Weighted proportion** % (95 CI)
**Age (mean, SE)**	45.39 (0.18)	-
**Age (categorized)**		
18 – 34 years	7 251	27.7 (26.8 – 28.6)
35 or more years	18 915	72.3 (71.4 – 73.2)
**Sex**		
Male	12 440	47.5 (46.4 – 48.6)
Female	13 726	52.5 (51.4 – 53.6)
**Area of residence**		
Rural	4 830	18.5 (17.9 – 19.0)
Urban	21 336	81.5 (81.0 – 82.1)
**Education level**		
No education	1 045	4.0 (3.6 – 4.4)
Incomplete elementary	4 126	15.8 (15.0 – 16.6)
Complete elementary	1 473	5.6 (5.2 – 6.0)
Incomplete secondary	3 332	12.7 (12.0 – 13.5)
Complete secondary	7 456	28.5 (27.5 – 29.5)
Higher education	8 734	33.4 (32.3 – 34.5)
**Ethnicity**		
Native	7 335	28.0 (27.1 – 29.0)
Zambo, afro descendant	2 998	11.5 (10.8 – 12.2)
White	1 842	7.0 (6.5 – 7.6)
Mestizo	12 349	47.2 (46.1 – 48.3)
Other	327	1.3 (1.0 – 1.5)
Does not know	1 315	5.0 (4.6 – 5.5)

**Economic level**		
The poorest	4 839	18.5 (17.8 – 19.2)
Poor	5 216	19.9 (19.1 – 20.8)
Middle	5 555	21.2 (20.3 – 22.2)
Rich	5 422	20.7 (19.7 – 21.8)
The richest	5 133	19.6 (18.5 – 20.8)
**Region of origin**		
Metropolitan Lima	9 789	37.4 (36.3 – 38.6)
Rest of the coast	6 848	26.2 (25.2 – 27.2)
Highlands	6 363	24.3 (23.3 – 25.4)
Jungle	3 166	12.1 (11.4 – 12.8)
**Altitude**		
Less than 3 000 m.a.s.l.	22 291	85.2 (84.3 – 86.1)
Great or equal to 3 000 m.a.s.l.	3 876	14,8 (13.9 – 15.7)
**Health insurance**		
Yes	21 539	82.3 (81.4 – 83.2)
No	4 627	17.7 (16.8 – 18.6)
**Hypertension**		
Present	2 982	11.4 (10.7 – 12.1)
Absent	23 184	88.6 (87.8 – 89.2)
**Daily smoker**		
No	25 780	98.5 (98.2 – 98.8)
Yes	386	1.5 (1.2 – 1.8)
**Alcohol drinking**		
Present	16 182	61.8 (60.7 – 62.9)
Absent	9 984	38.2 (37.1 – 39.3)

SE = Standard error, m.a.s.l. = meters above sea level

Prevalence of T2DM screening and associated factors

Among individuals who met the ADA T2DM screening criteria, only 25.3% had a blood sugar measurement in the last 12 months. Bivariate analysis (Table 2) revealed that screening was less common among the rural population (85.7%), individuals without formal education (80.5%) and those from lower socioeconomic levels. Screening prevalence was also lower in the highlands (80.9%), especially at an altitude exceeding 3,000 meters above sea level (83.1%). Additionally, individuals without health insurance (84.6%), those without a diagnosis of hypertension (77.3%), and daily smokers (83.1%) were less likely to be screened. Alcohol consumption in the last 30 days was not associated with screening prevalence (p = 0.7596).

Table 2. Factors associated with the lack of T2DM screening in the last 12 months: Bivariate analysis.

**Variables**	**Glucose testing in the last 12 months (N = 26 166)**

	No (n = 19546)	Yes (n = 6 620)	p*
	nº (%) 74.7%	nº (%) 25.3%	
**Age (categorized)**			
18 – 34 years	5 783 (79.8%)	1 468 (20.2%)	<0.001
35 or more years	13 769 (72.8%)	5 147 (27.2%)	
**Sex**			
Female	10 061 (73.3%)	3 666 (26.7%)	0.002
Male	9 490 (76.3%)	2 949 (23.7%)	
**Area of residence**			
Rural	4 140 (85.7%)	688 (14.3%)	<0.001
Urban	15 409 (72.2%)	5 927 (27.8%)	
**Education level**			
No education	843 (80.5%)	204 (19.5%)	<0.001
Incomplete elementary	3 297 (79.9%)	830 (20.1%)	
Complete elementary	1 287 (87.4%)	186 (12.6%)	
Incomplete secondary	2 601 (78.1%)	730 (21.9%)	
Complete secondary	5 730 (76.9%)	1 724 (23.1%)	
Higher education	5 793 (66.3%)	2 938 (33.7%)	
**Ethnicity**			
Native	5 694 (77.6%)	1 643 (22.4%)	<0.001
Zambo, afrodescendant	2 360 (78.7%)	639 (21.3%)	
White	1 376 (74.8%)	463 (25.2%)	
Mestizo	8 839 (71.6%)	3 512 (28.4%)	
Other	249 (75.9%)	79 (24.1%)	
Do not know/No response	1 034 (78.7%)	280 (21.3%)	
**Economic level**			
The poorest	4 210 (87.0%)	628 (13%)	<0.001
Poor	4 189 (80.3%)	1 028 (19.7%)	
Middle	4 228 (76.1%)	1 327 (23.9%)	
Rich	3 807 (70.2%)	1 614 (29.8%)	
The richest	3 116 (60.7%)	2 017 (39.3%)	
**Region of origin**			
Metropolitan Lima	6 863 (70.1%)	2 925 (29.9%)	<0.001
Rest of the coast	5 050 (73.8%)	1 798 (26.3%)	
Highlands	5 147 (80.9%)	1 217 (19.1%)	
Jungle	2 488 (78.6%)	678 (21.4%)	
**Altitude**			
Less than 3 000 m.a.s.l.	16 328 (73.2%)	5 963 (26.8%)	<0.001
Great or equal to 3 000 m.a.s.l.	3 224 (83.1%)	654 (16.9%)	
**Health insurance**			
Yes	15 637 (72.6%)	5 903 (27.4%)	<0.001
No	3 914 (84.6%)	712 (15.4%)	
**Hypertension**			
Present	1 622 (54.4%)	1 361 (45.6%)	<0.001
Absent	17 924 (77.3%)	5 259 (22.7%)	
**Daily smoker**			
No	319 (83.1%)	65 (16.9%)	0.0473
Yes	19 232 (74.6%)	6 549 (25.4%)	
**Alcohol drinking**			
Present	7 439 (74.5%)	2 543 (25.5%)	0.7596
Absent	12 110 (74.8%)	4 071 (25.2%)	

*p-value from chi-square test

Multivariate analysis identified several factors associated with the lack of screening (Fig 2). These were: age between 18-34 years (aPR: 1.08; 95%CI: 1.05-1.11), male sex (aPR: 1.00063; 95%CI: 1.00063-1.5), belonging to the lowest wealth quintile (aPR: 1.09; 95%CI: 1.05-1.13), having only elementary education (aPR: 1.18; 95%CI: 1.13-1.23), living at an altitude greater than 3,000 m.a.s.l (aPR: 1.04; 95%CI: 1.004-1.07), not having a health insurance (aPR: 1.15; 95%CI: 1.12-1.18), and being a daily smoker (aPR: 1.10; 95%CI: 1.01-1.19). Factors that increased the prevalence of screening included having hypertension (aPR: 0.73; 95%CI: 0.68-1.78) and being in the two highest wealth quintiles (fourth quintile aPR: 0.93; 95%CI: 0.90-0.97; fifth quintile aPR: 0.85; 95%CI: 0.81-0.89). No significant association was found for ethnicity, area of residence, or region of origin. Further details can be found in Table 3.

Table 3. Factors associated with the non-screening for T2DM: Multivariate analysis.

**Variables**	**Crude model**			**Adjusted model**		
	**PR**	**CI 95%**	**p**	**PR**	**CI 95%**	**p**
**Age**						
35 or more years	1.00			1.00		
18 – 34 years	1.10	1.07 - 1.12	<0.001	1.08	1.05 - 1.12	<0.001
**Sex**						
Female	1.00			1.00		
Male	1.04	1.01 - 1.07	0.002	1.03	1.00 - 1.05[a]	0.044
**Area of residence**						
Urban	1.00			1.00		
Rural	1.19	1.16 - 1.21	<0.001	1.01	0.99 - 1.04	0.320
**Education level**						
Higher education	1.00			1.00		
No education	1.21	1.15 - 1.29	<0.001	1.16	1.09 - 1.23	<0.001
Incomplete elementary	1.20	1.16 - 1.25	<0.001	1.15	1.09 - 1.20	<0.001
Complete elementary	1.32	1.26 - 1.37	<0.001	1.18	1.13 - 1.23	<0.001
Incomplete secondary	1.18	1.12 - 1.24	<0.001	1.11	1.05 - 1.16	<0.001
Complete secondary	1.16	1.11 - 1.20	<0.001	1.09	1.05 - 1.13	<0.001
**Ethnicity**						
Mestizo	1.00			1.00		
Native	1.08	1.05 - 1.12	<0.001	0.99	0.96 - 1.03	0.658
Zambo, afro descendant	1.10	1.06 - 1.14	<0.001	1.02	0.98 - 1.05	0.388
White	1.05	0.99 - 1.14	0.105	1.01	0.94 - 1.05	0.769
Other	1.06	0.95 - 1.19	0.318	1.01	0.90 - 1.14	0.971
**Economic level**						
Middle	1.00			1.00		
The poorest	1.14	1.11 - 1.18	<0.001	1.09	1.05 - 1.13	<0.001
Poor	1.06	1.02 - 1.09	0.001	1.03	0.99 - 1.06	0.078

Rich	0.92	0.88 - 0.96	<0.001	0.93	0.90 - 0.97	0.001
The richest	0.80	0.76 - 0.84	<0.001	0.85	0.81 - 0.89	<0.001
**Region of origin**						
Metropolitan Lima	1.00			1.00		
Rest of the coast	1.05	1.02 - 1.09	0.006	0.98	0.95 - 1.02	0.328
Highlands	1.15	1.12 - 1.19	<0.001	1.00	0.97 - 1.05	0.709
Jungle	1.12	1.08 - 1.16	<0.001	0.98	0.95 - 1.02	0.320
**Altitude**						
Less than 3 000 m.a.s.l.	1.00			1.00		
Great or equal to 3 000 m.a.s.l.	1.14	1.12 - 1.16	<0.001	1.04	1.00 - 1.07[b]	0.028
**Health insurance**						
Yes	1.00			1.00		
No	1.17	1.13 - 1.20	<0.001	1.15	1.12 - 1.18	<0.001
**Hypertension**						
Absent	1.00			1.00		
Present	0.71	0.66 - 0.75	<0.001	0.73	0.68 - 0.78	<0.001
**Daily smoker**						
No	1.00			1.00		
Yes	1.11	1.02 - 1.21	0.017	1.10	1.01 - 1.19	0.029

PR = Prevalence Ratio
Prevalence Ratios are calculated with non-screening as the dependent outcome
[a]Male Sex: 95%CI (1.00063 - 1.051108)
[b]Altitude Greater or equal to 3000 m.a.s.l.: 95%CI (1.003953 - 1.072157)

Fig 2. Forest Plot for Factors associated with the lack of screening for T2DM

Discussion

In this nationally representative analysis, only 25.3% of eligible individuals in Peru were screened for T2DM based on the 2022 ADA criteria. This prevalence is lower than estimates from previous national studies, such as 27.7% in 2020 (Trujillo-Minaya) and 30.6% in 2014 (Aedo) [10,11]. However, these studies did not apply updated ADA criteria and included broader age groups, which may explain these different estimates. To our knowledge, this is the first study in Peru to apply the revised ADA recommendations, which prioritize early detection.

Compared to other Latin American countries, screening rates in Peru are limited. In Mexico, 15.2% of adults accessed preventive diabetes services, while in Brazil, over 70% underwent glucose testing within the previous two years [12,13]. These disparities likely reflect differences in national screening protocols, primary care access, and methodological approaches.

Age was an associated factor for screening. Individuals aged 18–34 years were less likely to undergo screening, despite meeting ADA criteria. Previous studies report lower healthcare utilization in younger adults, who may have lower perceived susceptibility to chronic diseases [10,12,13–19]. This is concerning given the growing incidence of early-onset T2DM [20].

Additionally, men were less likely to be screened than women, consistent with evidence from multiple countries [10,14,21]. Differences in how men seek healthcare, along with work obligations and cultural norms, may explain this gap [16,19,22]. Similarly, individuals with lower educational level and those in the lowest wealth quintiles were less likely to be screened, likely due to disparities in health literacy, financial resources, and healthcare access [10,11,15,17].

Lack of health insurance was strongly associated with lower screening rates. This factor has been extensively described as a predictor for low healthcare utilization, and lower opportunities for screening [23–25]. In contrast, individuals with a prior diagnosis of hypertension were more likely to be screened, likely reflecting more frequent contact with healthcare providers and awareness of comorbidities [12,17,23]. Daily smoking was associated with reduced screening, aligning with previous findings that link unhealthy behaviors to lower use of preventive health services [12,17,26].

Although no significant difference was observed between urban and rural residence, individuals living at ≥3,000 meters above sea level had lower screening rates. This may reflect persistent geographical barriers to care in high-altitude Andean communities, where transportation challenges and limited healthcare infrastructure reduce access to routine services [27].

These findings highlight the need for targeted public health strategies to increase screening uptake, particularly among young adults, men, individuals with lower socioeconomic status, the uninsured, and those in geographically remote areas. Expanding access to primary care and community-based screening programs could help reduce disparities and improve early detection of T2DM.

In addition to addressing these gaps, adopting the ADA screening criteria in Peru may also represent a cost-effective strategy to enhance early detection. In the United States, the number needed to screen (NNS) is 15 for individuals aged ≥35 years and 12 for those aged ≥45 years, with estimated costs per positive case of $66.37 and $55.53, respectively [28]. While broader screening increases initial costs, it substantially reduces the number of missed diagnoses. Country-specific cost-effectiveness

analyses are needed to assess the feasibility and impact of implementing these recommendations in Peru.

This study has several strengths. It is based on a large, nationally representative sample drawn using a probabilistic design, enhancing generalizability. It is also the first to estimate T2DM screening coverage in Peru using the updated ADA criteria. Furthermore, it provides new insights into the sociodemographic and health-related determinants of screening in a middle-income setting.

However, limitations should be acknowledged. The cross-sectional design impedes causal inference. Recall bias may be present due to the self-reported nature of the data. The outcome of self-reported glucose testing in the past year may not always reflect screening for T2DM, as glucose may be tested in other clinical contexts. Additionally, the survey does not include information on other important predictors of screening, such as family history of diabetes. Finally, the lack of biomarker data prevents assessment of undiagnosed diabetes and limits estimation of a true screening gap.

Conclusion

Only 25.3% of the population that met the ADA diabetes screening criteria, underwent screening in Peru in 2022. The factors associated with the lack of screening included being in the age range of 18 to 34 years, being male, having a low level of education, belonging to the lower wealth quintiles, lacking health insurance, living at an altitude of 3,000 m.a.s.l or higher, and being a daily smoker. In this regard, efforts should be made to promote public policies that raise awareness about the importance of early diagnosis of T2DM and that target the population with these characteristics.

Acknowledgements

The authors acknowledge the help from Dr. Leandro Huayanay and Dr. Armando Pezo for initial statistical guidance.

References

1. Herman WH, Ye W, Griffin SJ, Simmons RK, Davies MJ, Khunti K, Rutten GEHM, Sandbaek A, Lauritzen T, Borch-Johnsen K, Brown MB, Wareham NJ. Early Detection and Treatment of Type 2 Diabetes Reduce Cardiovascular Morbidity and Mortality: A Simulation of the Results of the Anglo-Danish-Dutch Study of Intensive Treatment in People With Screen-Detected Diabetes in Primary Care (ADDITION-Europe). Diabetes Care. 2015 May 15;38(8):1449–55.
2. Nanayakkara N, Curtis AJ, Heritier S, Gadowski AM, Pavkov ME, Kenealy T, Owens DR, Thomas RL, Song S, Wong J, Chan JCN, Luk AOY, Penno G, Ji L, Mohan V, Amutha A, Romero-Aroca P, Gasevic D, Magliano DJ, Teede HJ, Chalmers J, Zoungas S. Impact of age at type 2 diabetes mellitus diagnosis on mortality and vascular complications: systematic review and meta-analyses. Diabetologia. 2021 Feb;64(2):275–87.
3. American Diabetes Association Professional Practice Committee. 2. Diagnosis and Classification of Diabetes: Standards of Care in Diabetes—2024. Diabetes Care. 2023 Dec 11;47(Supplement_1):S20–42.
4. Rios-Blancas MJ, Pando-Robles V, Razo C, Carcamo CP, Mendoza W, Pacheco-Barrios K, Miranda JJ, Lansingh VC, Demie TG, Saha M, Okonji OC, Yigit A, Cahuana-Hurtado L, Chacón-Uscamaita PR, Bernabe E, Culquichicon C, Chirinos-Caceres JL, Cárdenas R, Alcalde-Rabanal JE, Barrera FJ, Quintanilla BPA, Shorofi SA, Wickramasinghe ND, Ferreira N, Almidani L, Gupta VK, Karimi H, Alayu DS, Benziger CP, Fukumoto T, Mostafavi E, Redwan EMM, Gebrehiwot M, Khatab K, Koyanagi A, Krapp F, Lee S, Noori M, Qattea I, Rosenthal VD, Sakshaug JW, Wagaye B, Zare I, Ortega-Altamirano DV, Murillo-Zamora E, Vervoort D, Silva DAS, Oulhaj A, Herrera-Serna BY, Mehra R, Amir-Behghadami M, Adib N, Cortés S, Dang AK, Nguyen BT, Mokdad AH, Hay SI, Murray CJL, Lozano R, García PJ. Estimating mortality and disability in Peru before the COVID-19 pandemic: a systematic analysis from the Global Burden of the Disease Study 2019. Front Public Health. 2023 Jun 22;11:1189861.
5. Seclen SN, Rosas ME, Arias AJ, Huayta E, Medina CA. Prevalence of diabetes and impaired fasting glucose in Peru: report from PERUDIAB, a national urban population-based longitudinal study. BMJ Open Diabetes Res Care. 2015 Oct 1;3(1):e000110.
6. Instituto Nacional de Estadistica e Informatica. Peru: Enfermedades No Transmisibes y Transmisibles 2022 [Internet]. INEI; 2023 [cited 2024 Nov 4]. Available from: https://www.inei.gob.pe/media/MenuRecursivo/publicaciones_digitales/Est/Lib1951/libro.pdf
7. Ministerio de Salud del Peru. GUÍA DE PRÁCTICA CLÍNICA PARA EL DIAGNÓSTICO, TRATAMIENTO Y CONTROL DE LA DIABETES MELLITUS TIPO 2 EN EL PRIMER NIVEL DE ATENCIÓN [Internet]. 2016 [cited 2024 Nov 4]. Available from: https://bvs.minsa.gob.pe/local/MINSA/3466.pdf
8. Villena JE. Diabetes Mellitus in Peru. Ann Glob Health. 2015 Nov 1;81(6):765–75.
9. Gnardellis C, Notara V, Papadakaki M, Gialamas V, Chliaoutakis J. Overestimation of Relative Risk and Prevalence Ratio: Misuse of Logistic Modeling. Diagnostics. 2022 Nov 17;12(11):2851.
10. Trujillo-Minaya F, Vera-Ponce VJ, Torres-Malca JR, Zuzunaga-Montoya FE, Valencia JG, Cruz-Vargas JADL, Cruz-Ausejo L. Factores asociados al cribado de Diabetes Mellitus en población Peruana ¿problema para la salud pública? Rev Cuid [Internet]. 2023 Mar 15 [cited 2024 Nov 4];14(1). Available from: https://revistas.udes.edu.co/cuidarte/article/view/2792
11. Aedo Tito KP, Conde Bazán LF. Inequidades en el tamizaje para Diabetes Mellitus: estudio transversal de base poblacional en Perú, 2014. Repos Académico - UPC [Internet]. 2018 Jan 18 [cited 2024 Nov 4]; Available from: https://repositorioacademico.upc.edu.pe/handle/10757/622888
12. Villalobos A, Aguilar-Salinas CA, Martínez MR, Rojas-Martínez R. Perfiles poblacionales asociados con la asistencia a servicios preventivos para tamizaje de diabetes e hipertensión. Ensanut 2018-19. Salud Pública México. 2020 Nov 24;62(6, Nov-Dic):754–66.
13. Santos KBM, Reis RCP dos, Duncan BB, D'Avila OP, Schmidt MI. Access to diabetes diagnosis in Brazil based on recent testing and consultation: The Brazilian national health survey, 2013 and 2019. Front Endocrinol [Internet]. 2023 Mar 22 [cited 2024 Nov 4];14. Available from:

https://www.frontiersin.org/journals/endocrinology/articles/10.3389/fendo.2023.1122164/full
14. Dryden R, Williams B, McCowan C, Themessl-Huber M. What do we know about who does and does not attend general health checks? Findings from a narrative scoping review. BMC Public Health. 2012 Aug 31;12(1):723.
15. AshaRani PV, Devi F, Wang P, Abdin E, Zhang Y, Roystonn K, Jeyagurunathan A, Subramaniam M. Factors influencing uptake of diabetes health screening: a mixed methods study in Asian population. BMC Public Health. 2022 Aug 9;22(1):1511.
16. Teo CH, Ng CJ, White A. Factors influencing young men's decision to undergo health screening in Malaysia: a qualitative study. BMJ Open. 2017 Mar 10;7(3):e014364.
17. Paek SC, Zhang NJ. Factors associated with the use of annual health checkups in Thailand: evidence from a national cross-sectional health and welfare survey. Front Public Health [Internet]. 2024 May 10 [cited 2024 Nov 4];12. Available from: https://www.frontiersin.org/journals/public-health/articles/10.3389/fpubh.2024.1390125/full
18. Harvey IS, Alston RJ. Understanding preventive behaviors among mid-Western African-American men: a pilot qualitative study of prostate screening. J Mens Health. 2011 May 1;8(2):140.
19. Cobo B, Cruz C, Dick PC. Gender and racial inequalities in the access to and the use of Brazilian health services. Cienc Saude Coletiva. 2021 Sep;26(9):4021–32.
20. Strati M, Moustaki M, Psaltopoulou T, Vryonidou A, Paschou SA. Early onset type 2 diabetes mellitus: an update. Endocrine. 2024;85(3):965–78.
21. Kaul P, Chu LM, Dover DC, Yeung RO, Eurich DT, Butalia S. Disparities in adherence to diabetes screening guidelines among males and females in a universal care setting: A population-based study of 1,380,697 adults. Lancet Reg Health Am. 2022 Oct;14:100320.
22. Daniel-Ulloa J, Reyes JA, Morales-Campos DY, Villareal E, López Cevallos DF, Hernandez H, Baquero B. Rural Latino Men's Experiences and Attitudes Toward Health: A Pilot Photovoice Study. Am J Mens Health. 2023 Mar 9;17(2):15579883231158525.
23. Malta DC, Bernal RTI, Lima MG, Araújo SSC de, Silva MMA da, Freitas MI de F, Barros MB de A. Noncommunicable diseases and the use of health services: analysis of the National Health Survey in Brazil. Rev Saúde Pública. 2017 Jun 1;51:4s.
24. Mezones-Holguín E, Amaya E, Bellido-Boza L, Mougenot B, Murillo JP, Villegas-Ortega J, Sara JCDC. [Health insurance coverage: the peruvian case since the universal insurance act]. Rev Peru Med Exp Salud Publica. 2019;36(2):196–206.
25. Díaz-Ruiz R, Vargas-Fernández R, Rojas-Roque C, Hernández-Vásquez A. Socioeconomic inequalities in the use of medical consultation services in Peru, 2019. Int J Equity Health. 2024 Jan 20;23(1):10.
26. Thorogood M, Coulter A, Jones L, Yudkin P, Muir J, Mant D. Factors affecting response to an invitation to attend for a health check. J Epidemiol Community Health. 1993 Jun;47(3):224–8.
27. Sanchez-Samaniego G, Hartinger SM, Mäusezahl D, Hattendorf J, Fink G, Probst-Hensch N. Prevalence, awareness, treatment and control of high blood pressure in a cohort in Northern Andean Peru. Glob Health Action. 2023 Dec 1;16(1):2285100.
28. Ali MK, Imperatore G, Benoit SR, O'Brien MJ, Holliday CS, Echouffo-Tcheugui JB, McKeever Bullard K. Impact of changes in diabetes screening guidelines on testing eligibility and potential yield among adults without diagnosed diabetes in the United States. Diabetes Res Clin Pract. 2023 Mar;197:110572. doi: 10.1016/j.diabres.2023.110572. Epub 2023 Feb 11. PMID: 36775024; PMCID: PMC10352955.

Supporting information

S1 Data. Raw databases for Household information

S2 Data. Raw databases for Health information

S1 Table. Definition and Operationalization of Variables

Fig 1. Flowchart of participant selection

Click here to access/download;Figure;Fig1.tif

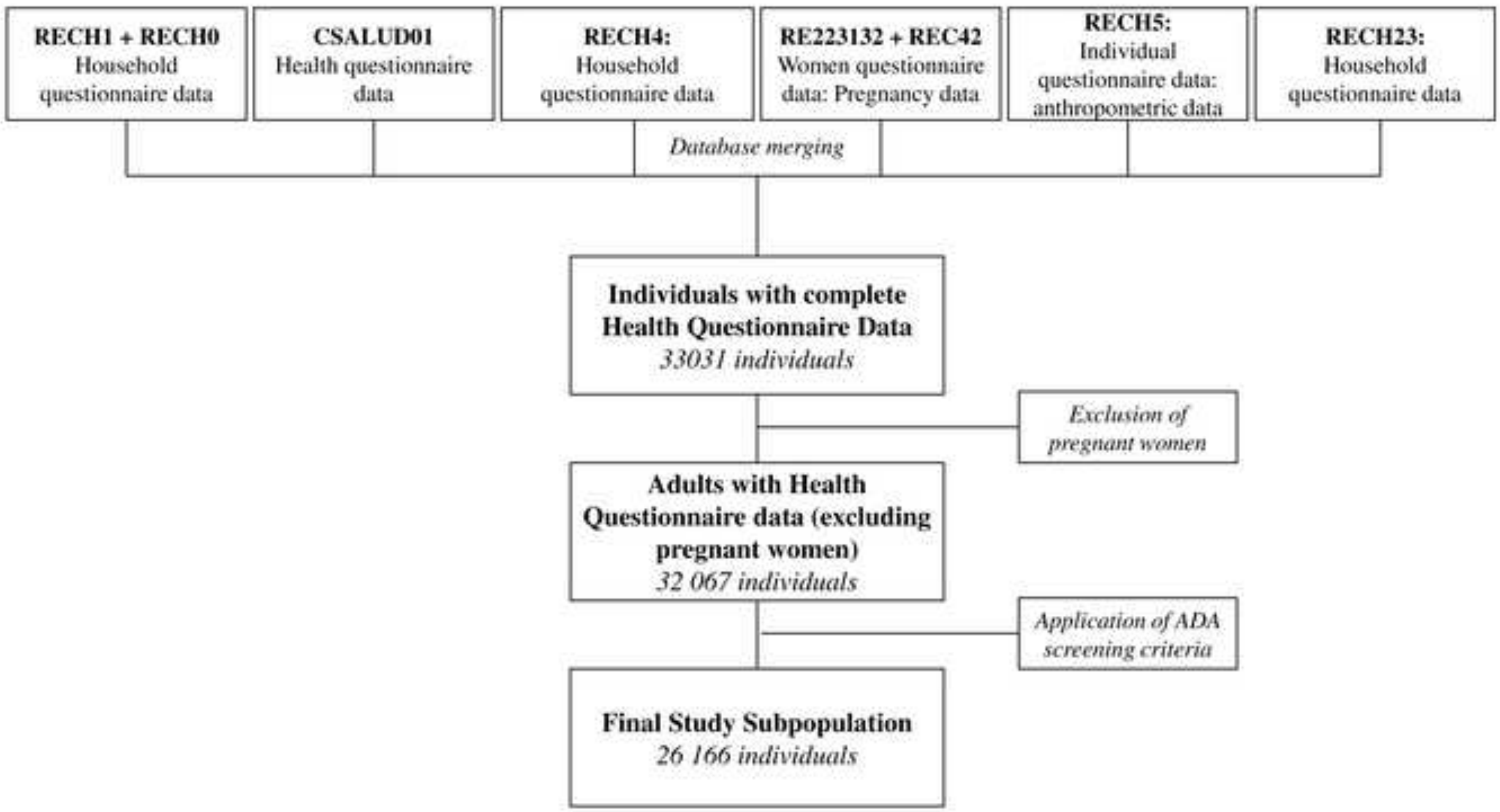

Fig 2. Forest Plot for Factors associated with the lack of screening for T2DM

Click here to access/download;Figure;Fig2.tif

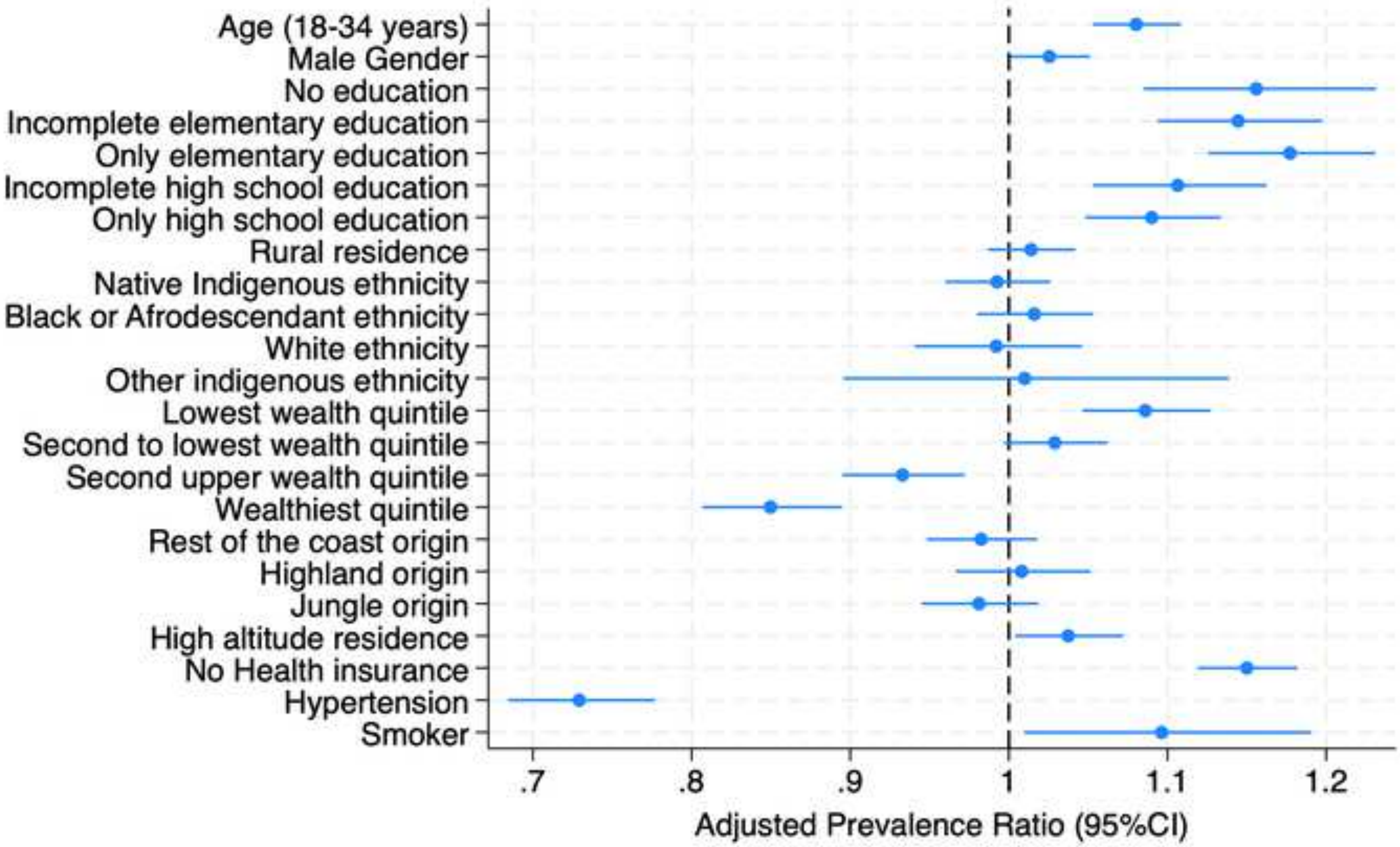

S1 Table. Definition and Operationalization of Variables

Click here to access/download

Supporting Information

S1_Table.docx

S1 Data. Raw databases for Household information

Click here to access/download
Supporting Information - Compressed/ZIP File Archive
S1_Data.zip

S2 Data. Raw databases for Health information

Click here to access/download
Supporting Information - Compressed/ZIP File Archive
S2_Data.zip

Diabetes Mellitus screening and associated factors in Peru: a cross-sectional analysis of a national health survey

Einer Carlos Eduardo Arevalo-Rios[1*], Jennifer Layza-Reyes [1], Victor Hugo Noriega-Ruiz [1,2]

[1] Alberto Hurtado School of Medicine, Universidad Peruana Cayetano Heredia, Lima, Peru

[2] Department of Endocrinology, Clinica Anglo Americana, Lima, Peru

* Corresponding author

E-mail: einer.arevalo@upch.pe (EA)

Abstract

Screening rates for Type 2 diabetes mellitus (T2DM) in Peru remain low despite an increasing prevalence of the disease. In 2022, the American Diabetes Association (ADA) recommended screening adults aged 18-34 with overweight or obesity and at least one risk factor, including Latino ethnicity and lowered the universal screening age to 35 years. ~~We~~ This study aimed to determine the T2DM screening prevalence in Peru and the factors associated with lack of screening in the population that meets ADA screening criteria. A cross-sectional, secondary data analysis was conducted based on the 2022 Peruvian Demographic and Health Survey database. Screening was defined as apparently healthy individuals having their blood glucose measured in the last year. Individuals aged 18-34 years overweight or obese and all individuals aged ≥ 35 years were included. Bivariate analysis was performed using the chi-squared test, and multivariate Poisson regression was used to estimate prevalence ratios (PRs), adjusting for potential confounders. Of 26,166 individuals who met inclusion criteria, 25.3% were screened for T2DM. The factors most strongly associated with lack of screening were: age 18-34 years (aPR: 1.08; 95% CI: 1.05-1.11), having only elementary education (aPR: 1.18; 95% CI: 1.13-1.23), not having health insurance (aPR: 1.15; 95% CI: 1.12-1.18), belonging to the lowest wealth quintile (aPR: 1.09; 95% CI: 1.05-1.13), and daily smoking (aPR: 1.10; 95% CI: 1.01-1.19). The national rate of screening for T2DM in Peru is low (25.3%). There is a higher prevalence for a lack of screening in people who: are younger, have only primary education, do not have health insurance, belong to the lowest wealth quintile, and are smokers. Targeted interventions are needed to improve screening coverage in these high-risk populations.

Introduction

~~Type 2~~ Diabetes Mellitus ~~(T2DM)~~ is one of the leading causes of mortality worldwide. However, early diagnosis and treatment can significantly reduce morbidity and mortality [1]. Longer periods without adequate metabolic control increase the risk of developing chronic complications that worsen prognosis [1,2]. Screening with a fasting plasma glucose measurement, glycated hemoglobin

(HbA1c), or glucose tolerance test is the main strategy for early detection of this disease. In 2022, the ~~American Diabetes Association (~~ADA~~)~~ modified its screening recommendations to include universal screening for all individuals starting at age 35; and targeted screening for individuals over 18 years old with overweight or obesity and at least one additional risk factor, including Latino ethnicity, which is considered high-risk [3]. These modifications were made to improve sensitivity of screening and to promote early detection of disease. Following these recommendations, and considering Peruvians as Latinos, all individuals with overweight or obesity between the ages of 18 and 34, as well as everyone over 35, should be screened for T2DM in Peru.

Peru is considered an upper-middle-income country with a geographically and ethnically diverse population that has persistent barriers to healthcare access despite recent economic growth [4]. Its diverse topography is marked by differences in the three main natural regions of the country: coast, highlands and jungle. Approximately 58% of the population resides on the coast, with many of the healthcare facilities concentrated in the capital districts of each department. The highlands of Peru are inhabited by 28.1% of the population, with many of them living at high altitudes, with limited infrastructures, deficient road networks and poor access to health services. The jungle of Peru, comprising 13% of the population, has many scattered native-indigenous communities with limited interconnection and many of them only accessible by air or water [4]. These characteristics pose an intrinsic barrier to healthcare access throughout the nation that impact early disease detection, including T2DM screening. Regarding non-communicable diseases, the prevalence of T2DM in Peru has increased in the past decades, with latest reports estimating a prevalence of 5-7 % highlighting variations among regions in the nation (coast, highlands, and jungle) [5,6]. ~~We hypothesize~~It is hypothesized that socioeconomic and cultural barriers affect the T2DM screening coverage. It is currently unknown how many people have been screened in the country following the latest ADA guideline recommendations. While the Ministry of Health in Peru has defined screening criteria in national guidelines, these have not been updated since 2016, despite the increasing prevalence of the disease in the country [5,7]. Since T2DM is a disease that leads to complications that can be prevented with timely diagnosis and treatment, ~~we~~ this study uses ~~believe that~~ the latest and more sensitive screening criteria ~~should be prioritized~~, which are the ADA screening recommendations. Therefore, in

this study ~~we used~~ data from a nationally representative health survey was analyzed to answer the following questions: What is the prevalence of diabetes screening in Peru, and what factors are associated with the lack of screening?

Materials and Methods

Study design and area

A cross-sectional analysis was performed using the 2022 Peruvian Demographic and Health Survey (DHS) database. This survey was conducted by the National Institute of Statistics and Informatics (INEI) from January to December 2022 on a nationally representative sample in all regions and departments of Peru. The DHS collected information through face-to-face interviews, and anthropometric measurements were obtained by trained individuals. The survey comprises multiple questionnaires; for this study, the Health Survey component was analyzed, as it includes data on non-communicable diseases such as T2DM~~This survey has different questionnaires; in this study, we utilized the Health Survey component, which collects data on non-communicable diseases such as T2DM~~. These datasets are publicly accessible at https://proyectos.inei.gob.pe/microdatos/ (S1-S2 Data).

Study population

The target population of the DHS are the usual residents of the interviewed households, which provide representative estimates of the population of Peru. In this study, ~~we analyzed~~ complete-case responses from those who met the 2022 ADA screening criteria were analyzed, considering Latino/Hispanic ethnicity as a risk factor. Body Mass Indexes were calculated based on the anthropometric measures obtained in the interviews and as recorded in the Health Survey.

Inclusion and Exclusion criteria

Participants were included based on the two key populations to screen according to the ADA:

- Adults aged 18-34 years with overweight or obesity and one additional risk factor, specifically Latino ethnicity, who had complete data in the Health Survey.
- All individuals aged 35 years and older with complete data in the Health Survey.

P~~We excluded p~~regnant women, individuals with a previous diagnosis of T2DM and those taking antidiabetic medications were excluded.

Data collection and sample size

The DHS used a two-stage, probabilistic, balanced, stratified, and independent sampling design, covering all regions of Peru. The Health Survey was administered to 36 650 households and interviewed 31 917 individuals aged 15 years and over. This study involved no direct contact with participants, as it was based on the analysis of publicly available datasets~~For this study, we did not have direct contact with the individuals and relied on the datasets~~. ~~We included only~~Only complete-case responses ~~of all individuals who met the inclusion criteria~~were analyzed.

Variables

The dependent variable of this study was the self-report of ~~diabetes mellitus~~T2DM screening; based on the question: “In the past 12 months, has a doctor or other health professional measured your blood glucose or “sugar” level?”. This outcome was categorized as a dichotomous variable.

Potential risk factors for the lack of screening were identified based on review of previous literature. These included age, sex, residence area, region of origin, altitude of residence, education level, ethnicity, wealth index and health insurance status. The wealth index was defined in quintiles as categorized in the DHS and based on a cumulative measure of a household’s living standard. Age was further categorized in 18-34 and 35 years or older to reflect the distinct screening group recommendations of the ADA. Altitude of residence was ~~further~~ categorized into living at an altitude

more than 3 000 meters above sea level (m.a.s.l.) or not, based on previous studies that have reported lower diabetes prevalence at higher altitude in Peru [8]. Clinical variables affecting screening rates were included: previous hypertension diagnosis, current alcohol consumption, and smoking status. Further description of variables included in the survey is included as supplementary information (S1 Table).

Statistical analysis

Data was analyzed using STATA SE (18.0 version, College Station, Texas 77845, USA). To account for the complex sampling design of the DHS and the analysis of a specific subpopulation, the *svy* and *subpop* commands were used. Raw databases were merged to include the sociodemographic, anthropometric and health questionnaire information of surveyed individuals. Categorical variables are reported as frequencies and proportions, while quantitative variables are reported as mean and standard error for normally distributed data. Chi-square tests were used for the bivariate analysis between the independent variables and the dependent variable. Multivariate analysis was conducted using generalized linear models with Robust Poisson regression variance estimation to calculate crude and adjusted prevalence ratios for non-screening, considering the expected outcome prevalence to be greater than 10% [9]. The variables included in the multivariate model were the ones that showed association in the bivariate model and those epidemiologically relevant according to the literature review. A p-value ≤ 0.05 was considered statistically significant.

Ethical considerations

Data from the DHS is available publicly online as published by the INEI. When conducting the field interviews, the interviewers obtained written and informed consent from each respondent before participating in the survey. For this study, ~~we accessed~~ data was accessed on June 26th, 2024. The information collected did not allow for the identification of individuals. As this is a secondary study and ~~we~~ no direct contact with participants was made~~did not have contact with the study population~~, informed consent was not required. The protocol of this study was reviewed and approved by the

Institutional Committee on Ethics in Human Research of the Peruvian University Cayetano Heredia in Lima, Peru.

Results

Characteristics of the study population~~Study population~~

The subpopulation that met the ADA screening criteria (35 years or older and 18-34 years with overweight or obesity) was 26,166 individuals (Fig 1). Among them, the majority ~~52.5%~~ were women~~,~~ (52.5%), over 35 years old, ~~81.5% lived~~resided in urban areas, identified as “Mestizo” ethnicity, and had health insurance. ~~33.4%~~Approximately one-third had achieved higher education~~, and 83.2% had health insurance~~. The distribution of the socioeconomic status categories was homogeneous, while ~~.~~ most lived in Metropolitan Lima. The majority did not smoke daily, 11.4% had been diagnosed with hypertension~~; 1.5% smoked daily~~, and ~~and 38.2% had consumed alcohol in the past 30 days.~~approximately one-third of individuals reported recent alcohol consumption in the past 30 days. The full description of the ~~sample~~ characteristics is shown in Table 1.

Fig 1. Flowchart of participant selection

Table 1. Characteristics of the population that met the ADA screening ~~and inclusion~~ criteria

Characteristics	**Total** (N= 26 166)	**Weighted proportion** % (95 CI)
**Age (mean, SE)**	45.39 (0.18)	-
**Age (categorized)**		
18 – 34 years	7 251	27.7 (26.8 – 28.6)
35 or more years	18 915	72.3 (71.4 – 73.2)
**Sex**		
Male	12 440	47.5 (46.4 – 48.6)
Female	13 726	52.5 (51.4 – 53.6)
**Area of residence**		
Rural	4 830	18.5 (17.9 – 19.0)
Urban	21 336	81.5 (81.0 – 82.1)
**Education level**		
No education	1 045	4.0 (3.6 – 4.4)
Incomplete elementary	4 126	15.8 (15.0 – 16.6)
Complete elementary	1 473	5.6 (5.2 – 6.0)
Incomplete secondary	3 332	12.7 (12.0 – 13.5)
Complete secondary	7 456	28.5 (27.5 – 29.5)

Higher education	8 734	33.4 (32.3 – 34.5)
**Ethnicity**		
Native	7 335	28.0 (27.1 – 29.0)
Zambo, afro descendant	2 998	11.5 (10.8 – 12.2)
White	1 842	7.0 (6.5 – 7.6)
Mestizo	12 349	47.2 (46.1 – 48.3)
Other	327	1.3 (1.0 – 1.5)
Does not know	1 315	5.0 (4.6 – 5.5)
**Economic level**		
The poorest	4 839	18.5 (17.8 – 19.2)
Poor	5 216	19.9 (19.1 – 20.8)
Middle	5 555	21.2 (20.3 – 22.2)
Rich	5 422	20.7 (19.7 – 21.8)
The richest	5 133	19.6 (18.5 – 20.8)
**Region of origin**		
Metropolitan Lima	9 789	37.4 (36.3 – 38.6)
Rest of the coast	6 848	26.2 (25.2 – 27.2)
Highlands	6 363	24.3 (23.3 – 25.4)
Jungle	3 166	12.1 (11.4 – 12.8)
**Altitude**		
Less than 3 000 m.a.s.l.	22 291	85.2 (84.3 – 86.1)
Great or equal to 3 000 m.a.s.l.	3 876	14,8 (13.9 – 15.7)
**Health insurance**		
Yes	21 539	82.3 (81.4 – 83.2)
No	4 627	17.7 (16.8 – 18.6)
**Hypertension**		
Present	2 982	11.4 (10.7 – 12.1)
Absent	23 184	88.6 (87.8 – 89.2)
**Daily smoker**		
No	25 780	98.5 (98.2 – 98.8)
Yes	386	1.5 (1.2 – 1.8)
**Alcohol drinking**		
Present	16 182	61.8 (60.7 – 62.9)
Absent	9 984	38.2 (37.1 – 39.3)

SE = Standard error, m.a.s.l. = meters above sea level

Prevalence of T2DM screening and associated factors

Among individuals who met the ADA T2DM screening criteria, only 25.3% had a blood sugar measurement in the last 12 months. Bivariate analysis (Table 2) revealed that screening was less common among the rural population (85.7%), individuals without formal education (80.5%) and those from lower socioeconomic levels. Screening prevalence was also lower in the highlands (80.9%), especially at an altitude exceeding 3,000 meters above sea level (83.1%). Additionally, individuals without health insurance (84.6%), those without a diagnosis of hypertension (77.3%), and daily smokers

(83.1%) were less likely to be screened. Alcohol consumption in the last 30 days was not associated with screening prevalence (p = 0.7596).

Table 2. Factors associated with the lack of T2DM screening in the last 12 months: Bivariate analysis.

Variables	Glucose testing in the last 12 months (N = 26 166)		
	No (n = 19546)	Yes (n = 6 620)	p*
	nº (%) 74.7%	nº (%) 25.3%	
**Age** (categorized)			
18 – 34 years	5 783 (79.8%)	1 468 (20.2%)	<0.001
35 or more years	13 769 (72.8%)	5 147 (27.2%)	
**Sex**			
Female	10 061 (73.3%)	3 666 (26.7%)	0.002
Male	9 490 (76.3%)	2 949 (23.7%)	
**Area of residence**			
Rural	4 140 (85.7%)	688 (14.3%)	<0.001
Urban	15 409 (72.2%)	5 927 (27.8%)	
**Education level**			
No education	843 (80.5%)	204 (19.5%)	<0.001
Incomplete elementary	3 297 (79.9%)	830 (20.1%)	
Complete elementary	1 287 (87.4%)	186 (12.6%)	
Incomplete secondary	2 601 (78.1%)	730 (21.9%)	
Complete secondary	5 730 (76.9%)	1 724 (23.1%)	
Higher education	5 793 (66.3%)	2 938 (33.7%)	
**Ethnicity**			
Native	5 694 (77.6%)	1 643 (22.4%)	<0.001
Zambo, afrodescendant	2 360 (78.7%)	639 (21.3%)	
White	1 376 (74.8%)	463 (25.2%)	
Mestizo	8 839 (71.6%)	3 512 (28.4%)	
Other	249 (75.9%)	79 (24.1%)	
Do not know/No response	1 034 (78.7%)	280 (21.3%)	
**Economic level**			
The poorest	4 210 (87.0%)	628 (13%)	<0.001
Poor	4 189 (80.3%)	1 028 (19.7%)	
Middle	4 228 (76.1%)	1 327 (23.9%)	
Rich	3 807 (70.2%)	1 614 (29.8%)	
The richest	3 116 (60.7%)	2 017 (39.3%)	
**Region of origin**			
Metropolitan Lima	6 863 (70.1%)	2 925 (29.9%)	<0.001
Rest of the coast	5 050 (73.8%)	1 798 (26.3%)	
Highlands	5 147 (80.9%)	1 217 (19.1%)	
Jungle	2 488 (78.6%)	678 (21.4%)	
**Altitude**			
Less than 3 000 m.a.s.l.	16 328 (73.2%)	5 963 (26.8%)	<0.001
Great or equal to 3 000 m.a.s.l.	3 224 (83.1%)	654 (16.9%)	
**Health insurance**			
Yes	15 637 (72.6%)	5 903 (27.4%)	<0.001
No	3 914 (84.6%)	712 (15.4%)	

Formatted: Centered

**Hypertension**			
Present	1 622 (54.4%)	1 361 (45.6%)	<0.001
Absent	17 924 (77.3%)	5 259 (22.7%)	
**Daily smoker**			
No	319 (83.1%)	65 (16.9%)	0.0473
Yes	19 232 (74.6%)	6 549 (25.4%)	
**Alcohol drinking**			
Present	7 439 (74.5%)	2 543 (25.5%)	0.7596
Absent	12 110 (74.8%)	4 071 (25.2%)	

*p-value from chi-square test

Multivariate analysis ~~using Poisson regression models to calculate crude (cPR) and adjusted prevalence ratios (aPR)~~ identified several factors associated with the lack of screening (Fig 2). These were: age between 18-34 years (aPR: 1.08; 95%CI: 1.05-1.11), male sex (aPR: 1.00063; 95%CI: 1.00063-1.5), belonging to the lowest wealth quintile (aPR: 1.09; 95%CI: 1.05-1.13), having only elementary education (aPR: 1.18; 95%CI: 1.13-1.23), living at an altitude greater than 3,000 m.a.s.l (aPR: 1.04; 95%CI: 1.004-1.07), not having a health insurance (aPR: 1.15; 95%CI: 1.12-1.18), and being a daily smoker (aPR: 1.10; 95%CI: 1.01-1.19). Factors that increased the prevalence of screening included having hypertension (aPR: 0.73; 95%CI: 0.68-1.78) and being in the two highest wealth quintiles (fourth quintile aPR: 0.93; 95%CI: 0.90-0.97; fifth quintile aPR: 0.85; 95%CI: 0.81-0.89). No significant association was found for ethnicity, area of residence, or region of origin. Further details can be found in Table 3.

Table 3. Factors associated with the non-screening for T2DM: Multivariate analysis.

Variables	Crude model			Adjusted model		
	PR	CI 95%	p	PR	CI 95%	p
**Age**						
35 or more years	1.00~~Ref.~~			1.00~~Ref.~~		
18 – 34 years	1.10	1.07 - 1.12	<0.001	1.08	1.05 - 1.12	<0.001
**Sex**						
Female	1.00~~Ref.~~			1.00~~Ref.~~		
Male	1.04	1.01 - 1.07	0.002	1.03	1.00 - 1.05[a]	0.044
**Area of residence**						
Urban	1.00~~Ref.~~			1.00~~Ref.~~		
Rural	1.19	1.16 - 1.21	<0.001	1.01	0.99 - 1.04	0.320
**Education level**						
Higher education	1.00~~Ref.~~			1.00~~Ref.~~		

Formatted: Right: 0"
Formatted: Centered
Formatted Table
Formatted: Centered
Formatted Table
Formatted: Centered
Formatted: Centered
Formatted Table
Formatted: Centered
Formatted: Centered
Formatted Table
Formatted: Centered
Formatted: Centered

No education	1.21	1.15 - 1.29	<0.001	1.16	1.09 - 1.23	<0.001
Incomplete elementary	1.20	1.16 - 1.25	<0.001	1.15	1.09 - 1.20	<0.001
Complete elementary	1.32	1.26 - 1.37	<0.001	1.18	1.13 - 1.23	<0.001
Incomplete secondary	1.18	1.12 - 1.24	<0.001	1.11	1.05 - 1.16	<0.001
Complete secondary	1.16	1.11 - 1.20	<0.001	1.09	1.05 - 1.13	<0.001
**Ethnicity**						
Mestizo	1.00~~Ref.~~			1.00~~Ref.~~		
Native	1.08	1.05 - 1.12	<0.001	0.99	0.96 - 1.03	0.658
Zambo, afro descendant	1.10	1.06 - 1.14	<0.001	1.02	0.98 - 1.05	0.388
White	1.05	0.99 - 1.14	0.105	1.01	0.94 - 1.05	0.769
Other	1.06	0.95 - 1.19	0.318	1.01	0.90 - 1.14	0.971
**Economic level**						
Middle	1.00~~Ref.~~			1.00~~Ref.~~		
The poorest	1.14	1.11 - 1.18	<0.001	1.09	1.05 - 1.13	<0.001
Poor	1.06	1.02 - 1.09	0.001	1.03	0.99 - 1.06	0.078
Rich	0.92	0.88 - 0.96	<0.001	0.93	0.90 - 0.97	0.001
The richest	0.80	0.76 - 0.84	<0.001	0.85	0.81 - 0.89	<0.001
**Region of origin**						
Metropolitan Lima	1.00~~Ref.~~			1.00~~Ref.~~		
Rest of the coast	1.05	1.02 - 1.09	0.006	0.98	0.95 - 1.02	0.328
Highlands	1.15	1.12 - 1.19	<0.001	1.00	0.97 - 1.05	0.709
Jungle	1.12	1.08 - 1.16	<0.001	0.98	0.95 - 1.02	0.320
**Altitude**						
Less than 3 000 m.a.s.l.	1.00~~Ref.~~			1.00~~Ref.~~		
Great or equal to 3 000 m.a.s.l.	1.14	1.12 - 1.16	<0.001	1.04	1.00 - 1.07[b]	0.028
**Health insurance**						
Yes	1.00~~Ref.~~			1.00~~Ref.~~		
No	1.17	1.13 - 1.20	<0.001	1.15	1.12 - 1.18	<0.001
**Hypertension**						
Absent	1.00~~Ref.~~			1.00~~Ref.~~		
Present	0.71	0.66 - 0.75	<0.001	0.73	0.68 - 0.78	<0.001
**Daily smoker**						
No	1.00~~Ref.~~			1.00~~Ref.~~		
Yes	1.11	1.02 - 1.21	0.017	1.10	1.01 - 1.19	0.029

PR = Prevalence Ratio~~, Ref. = Reference category~~

Prevalence Ratios are calculated with non-screening as the ~~main~~ dependent outcome

[a]Male Sex: ~~IC~~95%CI (1.00063 - 1.051108)

[b]Altitude ~~“~~Greater or equal to 3000 m.a.s.l.: ~~IC~~95%CI (1.003953 - 1.072157)

Fig 2. Forest Plot for Factors associated with the lack of screening for T2DM

Discussion

In this nationally representative analysis, ~~we found that ~~only 25.3% of eligible individuals in Peru were screened for ~~type 2 diabetes mellitus (~~T2DM~~)~~ ~~according ~~based on ~~to ~~the 2022 ADA criteria. This prevalence is lower than ~~that reported~~estimates from ~~in ~~previous national studies, such as 27.7% in 2020 (Trujillo-Minaya) and 30.6% in 2014 (Aedo)~~.~~ ~~Trujillo-Minaya reported a screening prevalence of 27.7% using 2020 DHS data, and Aedo reported 30.6% based on the 2014 DHS ~~[10,11]. However, these ~~earlier ~~studies did not apply updated ADA ~~screening ~~criteria and included broader age groups, ~~or older populations, ~~which may explain these ~~higher ~~different estimates. To our knowledge, this is the first study in Peru to apply the revised ADA ~~guidelines~~recommendations, which prioritize early detection~~, which were modified to increase case detection~~.

Compared to other Latin American countries, screening rates in Peru ~~remain ~~are ~~low~~limited. In Mexico, ~~only ~~15.2% of adults accessed preventive diabetes services, while in Brazil, over 70% underwent glucose testing within the previous two years [12,13]. These disparities likely reflect differences in national screening protocols, primary care access, and methodological approaches.

Age was an associated factor for screening. Individuals aged 18–34 years were less likely to undergo screening, despite ~~being eligible under~~meeting ADA criteria. ~~ if they are overweight or obese and have additional risk factors such as Latino ethnicity. Prior~~Previous studies ~~also ~~report lower healthcare utilization in younger adults, who may have lower perceived susceptibility to chronic diseases [10,12,13–19]. This is concerning given the growing incidence of early-onset T2DM [20].

~~We also found that~~Additionally, men were less likely to be screened than women, consistent with evidence from ~~Peru and other~~multiple countries [10,14,21]. Differences in how men seek healthcare, along with work obligations and cultural norms, may explain this gap ~~Gender-related differences in health-seeking behavior, as well as work-related and sociocultural barriers, may contribute to this gap ~~[16,19,22]. Similarly, individuals with lower educational ~~attainment ~~level and those in the lowest wealth quintiles were less likely to be screened, likely due to disparities in health literacy, financial resources, and healthcare access [10,11,15,17].

Lack of health insurance was strongly associated with lower screening rates. This factor has been extensively described as a predictor for low healthcare utilization, and lower opportunities for screening ~~Despite an increase in insurance coverage in Peru—reaching 76% of the population by 2017—engagement with preventive services remains suboptimal~~ [23–25]. In contrast, individuals with a prior diagnosis of hypertension were more likely to be screened, likely reflecting more frequent contact with healthcare providers and awareness of ~~comorbid~~comorbidities ~~risks~~ [12,17,23]. Daily smoking~~, on the other hand,~~ was associated with reduced screening, ~~consistent with prior literature linking~~ aligning with previous findings that link unhealthy behaviors to lower use of preventive health services [12,17,26].

~~While~~ Although no significant difference was observed between urban and rural residence, individuals living at ≥3,000 meters above sea level had lower screening rates. This may reflect persistent geographical barriers to care in high-altitude Andean communities, where transportation challenges and limited healthcare infrastructure reduce access to routine services [27].

These findings highlight the need for targeted public health strategies to increase screening uptake, particularly among young adults, men, individuals with lower socioeconomic status, the uninsured, and those in geographically remote areas.~~. Aligning national guidelines with ADA criteria and~~ E~~e~~xpanding access to primary care and community-based screening programs could help reduce disparities and improve early detection of T2DM.

In addition to addressing these gaps, a~~A~~dopting the ADA screening criteria in Peru may also represent a cost-effective strategy to enhance early detection. In the United States, the number needed to screen (NNS) is 15 for individuals aged ≥35 years and 12 for those aged ≥45 years, with estimated costs per positive case of $66.37 and $55.53, respectively [28]. While broader screening increases initial costs, it substantially reduces the number of missed diagnoses. Country-specific cost-effectiveness analyses are needed to assess the feasibility and impact of implementing these recommendations in Peru.

This study has several strengths. It is based on a large, nationally representative sample drawn using a probabilistic design, enhancing generalizability. It is also the first to estimate T2DM screening coverage in Peru using the updated ADA criteria. Furthermore, it provides new insights into the

sociodemographic and health-related determinants of screening in a middle-income setting~~First, it draws on a large, nationally representative sample obtained through a probabilistic sampling design, enhancing generalizability. Second, to our knowledge, it is the first study to estimate T2DM screening prevalence in Peru using the updated ADA criteria. Third, it is among the few studies in Peru to examine a range of health and sociodemographic determinants of preventive care for non-communicable diseases~~.

However, ~~we also acknowledge~~ limitations should be acknowledged. The cross-sectional design impedes causal inference. Recall bias may be present due to the self-reported nature of the data. The outcome of self-reported glucose testing in the past year may not always reflect screening for T2DM, as glucose may be tested in other clinical contexts. Additionally, the survey does not include information on other important predictors of screening, such as family history of diabetes. ~~Nevertheless, it remains the best feasible approach wih the current database.~~ Finally, the lack of biomarker data prevents assessment of undiagnosed diabetes and limits estimation of a true screening gap.

Conclusion

Only 25.3% of the population that met the ADA diabetes screening criteria, underwent screening in Peru in 2022. The factors associated with the lack of screening included being in the age range of 18 to 34 years, being male, having a low level of education, belonging to the lower wealth quintiles, lacking health insurance, living at an altitude of 3,000 m.a.s.l or higher, and being a daily smoker. In this regard, efforts should be made to promote public policies that raise awareness about the importance of early diagnosis of T2DM and that target the population with ~~the aforementioned~~ these characteristics.

Acknowledgements

The authors~~We~~ acknowledge the help from Dr. Leandro Huayanay and Dr. Armando Pezo for initial statistical guidance.

References

1. Herman WH, Ye W, Griffin SJ, Simmons RK, Davies MJ, Khunti K, Rutten GEHM, Sandbaek A, Lauritzen T, Borch-Johnsen K, Brown MB, Wareham NJ. Early Detection and Treatment of Type 2 Diabetes Reduce Cardiovascular Morbidity and Mortality: A Simulation of the Results of the Anglo-Danish-Dutch Study of Intensive Treatment in People With Screen-Detected Diabetes in Primary Care (ADDITION-Europe). Diabetes Care. 2015 May 15;38(8):1449–55.
2. Nanayakkara N, Curtis AJ, Heritier S, Gadowski AM, Pavkov ME, Kenealy T, Owens DR, Thomas RL, Song S, Wong J, Chan JCN, Luk AOY, Penno G, Ji L, Mohan V, Amutha A, Romero-Aroca P, Gasevic D, Magliano DJ, Teede HJ, Chalmers J, Zoungas S. Impact of age at type 2 diabetes mellitus diagnosis on mortality and vascular complications: systematic review and meta-analyses. Diabetologia. 2021 Feb;64(2):275–87.
3. American Diabetes Association Professional Practice Committee. 2. Diagnosis and Classification of Diabetes: Standards of Care in Diabetes—2024. Diabetes Care. 2023 Dec 11;47(Supplement_1):S20–42.
4. Rios-Blancas MJ, Pando-Robles V, Razo C, Carcamo CP, Mendoza W, Pacheco-Barrios K, Miranda JJ, Lansingh VC, Demie TG, Saha M, Okonji OC, Yigit A, Cahuana-Hurtado L, Chacón-Uscamaita PR, Bernabe E, Culquichicon C, Chirinos-Caceres JL, Cárdenas R, Alcalde-Rabanal JE, Barrera FJ, Quintanilla BPA, Shorofi SA, Wickramasinghe ND, Ferreira N, Almidani L, Gupta VK, Karimi H, Alayu DS, Benziger CP, Fukumoto T, Mostafavi E, Redwan EMM, Gebrehiwot M, Khatab K, Koyanagi A, Krapp F, Lee S, Noori M, Qattea I, Rosenthal VD, Sakshaug JW, Wagaye B, Zare I, Ortega-Altamirano DV, Murillo-Zamora E, Vervoort D, Silva DAS, Oulhaj A, Herrera-Serna BY, Mehra R, Amir-Behghadami M, Adib N, Cortés S, Dang AK, Nguyen BT, Mokdad AH, Hay SI, Murray CJL, Lozano R, García PJ. Estimating mortality and disability in Peru before the COVID-19 pandemic: a systematic analysis from the Global Burden of the Disease Study 2019. Front Public Health. 2023 Jun 22;11:1189861.
5. Seclen SN, Rosas ME, Arias AJ, Huayta E, Medina CA. Prevalence of diabetes and impaired fasting glucose in Peru: report from PERUDIAB, a national urban population-based longitudinal study. BMJ Open Diabetes Res Care. 2015 Oct 1;3(1):e000110.
6. Instituto Nacional de Estadistica e Informatica. Peru: Enfermedades No Transmisibes y Transmisibles 2022 [Internet]. INEI; 2023 [cited 2024 Nov 4]. Available from: https://www.inei.gob.pe/media/MenuRecursivo/publicaciones_digitales/Est/Lib1951/libro.pdf
7. Ministerio de Salud del Peru. GUÍA DE PRÁCTICA CLÍNICA PARA EL DIAGNÓSTICO, TRATAMIENTO Y CONTROL DE LA DIABETES MELLITUS TIPO 2 EN EL PRIMER NIVEL DE ATENCIÓN [Internet]. 2016 [cited 2024 Nov 4]. Available from: https://bvs.minsa.gob.pe/local/MINSA/3466.pdf
8. Villena JE. Diabetes Mellitus in Peru. Ann Glob Health. 2015 Nov 1;81(6):765–75.
9. Gnardellis C, Notara V, Papadakaki M, Gialamas V, Chliaoutakis J. Overestimation of Relative Risk and Prevalence Ratio: Misuse of Logistic Modeling. Diagnostics. 2022 Nov 17;12(11):2851.
10. Trujillo-Minaya F, Vera-Ponce VJ, Torres-Malca JR, Zuzunaga-Montoya FE, Valencia JG, Cruz-Vargas JADL, Cruz-Ausejo L. Factores asociados al cribado de Diabetes Mellitus en población Peruana ¿problema para la salud pública? Rev Cuid [Internet]. 2023 Mar 15 [cited 2024 Nov 4];14(1). Available from: https://revistas.udes.edu.co/cuidarte/article/view/2792
11. Aedo Tito KP, Conde Bazán LF. Inequidades en el tamizaje para Diabetes Mellitus: estudio transversal de base poblacional en Perú, 2014. Repos Académico - UPC [Internet]. 2018 Jan 18 [cited 2024 Nov 4]; Available from: https://repositorioacademico.upc.edu.pe/handle/10757/622888
12. Villalobos A, Aguilar-Salinas CA, Martínez MR, Rojas-Martínez R. Perfiles poblacionales asociados con la asistencia a servicios preventivos para tamizaje de diabetes e hipertensión. Ensanut 2018-19. Salud Pública México. 2020 Nov 24;62(6, Nov-Dic):754–66.
13. Santos KBM, Reis RCP dos, Duncan BB, D'Avila OP, Schmidt MI. Access to diabetes diagnosis in Brazil based on recent testing and consultation: The Brazilian national health survey, 2013 and 2019. Front Endocrinol [Internet]. 2023 Mar 22 [cited 2024 Nov 4];14. Available from:

https://www.frontiersin.org/journals/endocrinology/articles/10.3389/fendo.2023.1122164/full
14. Dryden R, Williams B, McCowan C, Themessl-Huber M. What do we know about who does and does not attend general health checks? Findings from a narrative scoping review. BMC Public Health. 2012 Aug 31;12(1):723.
15. AshaRani PV, Devi F, Wang P, Abdin E, Zhang Y, Roystonn K, Jeyagurunathan A, Subramaniam M. Factors influencing uptake of diabetes health screening: a mixed methods study in Asian population. BMC Public Health. 2022 Aug 9;22(1):1511.
16. Teo CH, Ng CJ, White A. Factors influencing young men's decision to undergo health screening in Malaysia: a qualitative study. BMJ Open. 2017 Mar 10;7(3):e014364.
17. Paek SC, Zhang NJ. Factors associated with the use of annual health checkups in Thailand: evidence from a national cross-sectional health and welfare survey. Front Public Health [Internet]. 2024 May 10 [cited 2024 Nov 4];12. Available from: https://www.frontiersin.org/journals/public-health/articles/10.3389/fpubh.2024.1390125/full
18. Harvey IS, Alston RJ. Understanding preventive behaviors among mid-Western African-American men: a pilot qualitative study of prostate screening. J Mens Health. 2011 May 1;8(2):140.
19. Cobo B, Cruz C, Dick PC. Gender and racial inequalities in the access to and the use of Brazilian health services. Cienc Saude Coletiva. 2021 Sep;26(9):4021–32.
20. Strati M, Moustaki M, Psaltopoulou T, Vryonidou A, Paschou SA. Early onset type 2 diabetes mellitus: an update. Endocrine. 2024;85(3):965–78.
21. Kaul P, Chu LM, Dover DC, Yeung RO, Eurich DT, Butalia S. Disparities in adherence to diabetes screening guidelines among males and females in a universal care setting: A population-based study of 1,380,697 adults. Lancet Reg Health Am. 2022 Oct;14:100320.
22. Daniel-Ulloa J, Reyes JA, Morales-Campos DY, Villareal E, López Cevallos DF, Hernandez H, Baquero B. Rural Latino Men's Experiences and Attitudes Toward Health: A Pilot Photovoice Study. Am J Mens Health. 2023 Mar 9;17(2):15579883231158525.
23. Malta DC, Bernal RTI, Lima MG, Araújo SSC de, Silva MMA da, Freitas MI de F, Barros MB de A. Noncommunicable diseases and the use of health services: analysis of the National Health Survey in Brazil. Rev Saúde Pública. 2017 Jun 1;51:4s.
24. Mezones-Holguín E, Amaya E, Bellido-Boza L, Mougenot B, Murillo JP, Villegas-Ortega J, Sara JCDC. [Health insurance coverage: the peruvian case since the universal insurance act]. Rev Peru Med Exp Salud Publica. 2019;36(2):196–206.
25. Díaz-Ruiz R, Vargas-Fernández R, Rojas-Roque C, Hernández-Vásquez A. Socioeconomic inequalities in the use of medical consultation services in Peru, 2019. Int J Equity Health. 2024 Jan 20;23(1):10.
26. Thorogood M, Coulter A, Jones L, Yudkin P, Muir J, Mant D. Factors affecting response to an invitation to attend for a health check. J Epidemiol Community Health. 1993 Jun;47(3):224–8.
27. Sanchez-Samaniego G, Hartinger SM, Mäusezahl D, Hattendorf J, Fink G, Probst-Hensch N. Prevalence, awareness, treatment and control of high blood pressure in a cohort in Northern Andean Peru. Glob Health Action. 2023 Dec 1;16(1):2285100.
28. Ali MK, Imperatore G, Benoit SR, O'Brien MJ, Holliday CS, Echouffo-Tcheugui JB, McKeever Bullard K. Impact of changes in diabetes screening guidelines on testing eligibility and potential yield among adults without diagnosed diabetes in the United States. Diabetes Res Clin Pract. 2023 Mar;197:110572. doi: 10.1016/j.diabres.2023.110572. Epub 2023 Feb 11. PMID: 36775024; PMCID: PMC10352955.

Supporting information

S1 Data. Raw databases for Household information

S2 Data. Raw databases for Health information

S1 Table. Definition and Operationalization of Variables

July 14, 2025

Re: Diabetes Mellitus screening and associated factors in Peru: a cross-sectional analysis of a national health survey

Einer Carlos Eduardo Arevalo-Rios, M.D., Jennifer Layza-Reyes, M.D., Victor Hugo Noriega-Ruiz, M.D.

Dear Editorial Board of PLOS Global Public Health,

We are very appreciative of the careful revision for our manuscript. We have addressed each of the points raised in this round of revision. Specifically, changes include additional proofreading of the discussion and minor changes in table format and tone. In the section that follows we have indicated our direct responses to each of the questions raised by your thorough review. In addition, we are attaching the version with tracked changes and an unmarked version of the manuscript. We believe the manuscript is significantly improved because of your observations and hope that you will find the revised version of this manuscript acceptable.

RESPONSE TO REVIEWER #1

General comments:

o please don't use pronouns at all (Like (we))

Thank you for this feedback. We have switched to use the third person in the article and eliminated first person pronouns from the text.

o define the abbreviation when used at first time, then use the symbol.

Thank you for pointing this out. The abbreviations are now defined the first time they are introduced in the article (including abstract). After its definition, only symbols are used.

o please describe the study population to general idea of the study participants, the author many not need to account the per-centages.

Thank you for this suggestion. We have implemented this recommendation: the study population section describes a general idea of participants, without excessive percentages. We have also re-titled this section in results to "Characteristics of the study population", as we noted that "study population" was repeated in the methods section and could lead to confusion (manuscript page 7).

o better to add variable in the description of study population.

UNIVERSIDAD PERUANA
CAYETANO HEREDIA

Thank you for this perspective. We have added variables that were missing in the description of study population, mainly age group, area of residence and ethnicity (manuscript page 7).

o describe how the author applied the multivariate analysis in the current data. and avoid to write about the results.
Thank you for this comment. The details of how the multivariate analysis was performed are in the Statistical Analysis subsection of the Methods (manuscript page 6). In the Results section (manuscript page 10), we have revised the text to focus exclusively on reporting the findings from the multivariate analysis, without restating methodological details. We have kept the narrative description of the multivariate analysis results, as we consider the written description important for the readers to understand the most associated factors.

o inclusion criteria was already mentioned. table 1: characteristics of the population.
Thank you for this observation, we have removed this from the Table 1 title.

o it is possible to find bivariate analysis using chi squire test to analyze age ?
Thank you for this concern. For the bivariate and multivariate analysis, we categorized age as a dichotomous categorical variable based on the ADA screening populations: Younger age 18-34 (1) and 35 and older (2). We used the chi-square test in this situation because we dealt with categorized variables. We have clarified this in Table 2 as "Age (categorized)".

o author can use different method for layout to avoid repartition of (ref)
Thank you for pointing this out. We have adjusted the format of Table 3 to avoid repetition of "Ref", and now show the Reference category with a Prevalence Ratio (PR) of 1.00.

o discussion need reread to rewrite in easy, intelligent, have high quality and integrity in language and ideas.
Thank you for your attention to this. We have proofread the discussion and adapted the language to be precise and easier to read. We hope this is now clearer.

RESPONSE TO REVIEWER #2

Thanks for the revision and responses. I have no further comments.

Thank you for revising our manuscript.
