# Supplementary material for: Diabetes Mellitus screening and associated factors in Peru: A cross-sectional analysis of a national health survey
Source: PLOS Glob Public Health. 2025 Sep 3;5(9):e0005150. doi: 10.1371/journal.pgph.0005150 (PMC12407462; doi:10.1371/journal.pgph.0005150)
Supplement: Attachment — Submitted filename: Response to Reviewers.docx [file pgph.0005150.s005.docx]

**June 21, 2025**

**Re: Diabetes Mellitus screening and associated factors in Peru: a cross-sectional analysis of a national health survey**

**Einer Carlos Eduardo Arevalo-Rios, M.D., Jennifer Layza-Reyes, M.D., Victor Hugo Noriega-Ruiz, M.D.**

Dear Editorial Board of PLOS Global Public Health,

We are very appreciative of the careful reading for our manuscript. We meticulously addressed each of the points and questions raised and performed the corrections or justifications in accordance. In the discussion that follows we have indicated our direct responses to each of the questions raised by your thorough review. We believe the manuscript is significantly improved because of your constructive criticisms and hope that you will find the revised version of this manuscript acceptable.

**RESPONSE TO REVIEWER #1**

**1. Good and important topic.**

Thank you for this comment.

**2. The authors need great effort to represent this topic in scientific manner.**

We appreciate this suggestion. We have revised and modified the writing style, terminology and tone of the article to align with the scientific nature of the journal.

**3. The author need to:
-rearrange idea specially the objective and,
- precise selection for factors and variables of the recent study**

Thank you for your insightful comment. We would like to reiterate the aim of our study and clarify the reason for including individuals under 35 years of age. Our objective was to estimate the prevalence of diabetes mellitus (DM) screening in Peru according to the 2022 American Diabetes Association (ADA) guidelines. These updated guidelines recommend screening in two key populations:

1. Adults aged 18–34 years with overweight or obesity and at least one additional risk factor; and
2. All adults aged 35 years or older, regardless of risk factors.

Latino ethnicity, which encompasses the Peruvian population, is explicitly listed as a risk factor by the ADA. Therefore, overweight or obese individuals aged 18–34 in Peru meet the ADA criteria and should be screened. Based on this, their inclusion in the analysis was justified. We have revised the manuscript to explicitly address this point in the Abstract, Study Population and Inclusion Criteria sections (manuscript page 4-5).

Additionally, the variables examined in our analysis were selected based on a review of literature on barriers to screening for non-communicable diseases. Corresponding items were then identified within the ENDES survey to operationalize these factors.

**Abstract:
4. need some details that belong to individuals who makes blood screen if:
-healthy
-apparently healthy
-have one or more than one of communicable disease
-have any health problem**

Thank you for the opportunity to clarify this. All adults 18 years or older were asked if they had their glucose measured in the last year, regardless of disease status.We selected the population 18 years or older that had not been diagnosed with DM (apparently healthy individuals), in order to comply with the principle of screening. We have added this in the abstract (manuscript page 2).

**-25.3% from the total number of diabetic who were screened as diabetic in Peru or according to the the research criteria?**

Thank you for this feedback. We modified this sentence to reflect this percentage belongs to those who met inclusion criteria (manuscript page 2). However, we would like to emphasize the importance of the DHS in that its sample provides nationally representative estimates.

**Introduction:
5. Fine.**

Thank you for this comment.

**Methodology:
6. what is ENDES? please define all the abbreviations.**

Thank you for pointing this out. We have uniformized the abbreviations to show them in English. ENDES initially referred to the Spanish translation of the “Demographic and Health Survey” (DHS). We have switched to using “DHS” in all the article.

**7. the researchers make questioner with self interview with participants? or from registered data of ENDES?**

We added clarification for this in the methods - data collection section (manuscript page 5). We relied on previously registered data from the DHS. We did not perform any interviews with participants for this study.

**8. Variables: the authors need to categorize materials and methods in subtitles include:**
**--study design, area
- study population
- inclusion and exclusion criteria of participants.
-method of sample and data collection
-ethical consideration
-methods for data analysis
*summarize methodology by avoiding repeating of information.
*the personal and anthropometric and clinical data which obtained in the the questioner are discussed in intelligent way in methods and results.
*return to and view scientific papers.**

We appreciate this feedback and have reorganized the methods section to include all these subsections (manuscript pages 4-6). We have avoided repetition of information.

**9. Ethics: what about the questioner of the study and participants?**

Thank you for this observation. We have added that the DHS interviewers obtained written and informed consent for each surveyed individual (manuscript page 6).

**Results:
10. Table 1: researchers must show only the descriptive criteria of the study population(selected participants)**

Thank you for this observation. We have revised the table to only include descriptive criteria. Accordingly, we have moved the glucose testing frequency (screening) to now only show in Table 2, as two columns (manuscript page 8).

**11. Data analysis is not meet the ADA T2DM criteria as the author mentioned for example age.**

We would like to offer clarification. As we mentioned in the previous response, the ADA criteria includes individuals 18-34 who are obese and from Latino ethnicity; additionally to everyone 35 years and older. That is why we included this age range.

**12. Table 2: -the total number of the participants in table 1 was 26166 and in table 2, 19546 participants.**

We appreciate this observation. Table 2 is divided into those not screened (19 546 individuals) and those screened (6 620 individuals), totaling 26 166 individuals, which is the same total number shown in Table 1. We have adapted Table 2 to show the total N (26 166) on top. This ensures both tables are in accordance (manuscript page 8-9).

**13. the researcher use the age group (>=35 years) and other specific inclusion criteria, data analysis must be adjusted to the objectives of the study.**

Thank you for this observation. We respectfully clarify that the age-based inclusion criteria used in our analysis are aligned with the study’s objective to estimate the prevalence of diabetes screening in Peru according to the ADA guidelines. These guidelines recommend universal screening for individuals aged ≥35 years, and targeted screening for individuals aged 18–34 years with overweight or obesity and at least one additional risk factor, including Latino ethnicity. Based on this, we included both groups as they are eligible for screening under the current recommendations.

**14. Table 3: this table may change as data entering change.**

Thank you for this concern, we have revised the analysis, and the results remain the same as the initial ones given the clarification on the included population for the study, and the confirmation that Table 1 and Table 2 include the same number of individuals.

**Discussion:
15. please discuss the results of current study and give justification of of results then compare them with previous and the more recent and similar studies,. in not more that one page.
-better to discuss directly the results only in brief, precise and scientific language.
-avoid repeating of data.
in discussion of current study the researcher open new windows which are not included in the objective of the study or calculated as variables in the data analysis.
-the authors discuss an important factor which is not included in the factors associated with the decrease screening in Peru.
-by the end of discussion you can add limitations and recommendation in points.
-acknowledgement is needed for any one who assist in this research.**

Thank you for these suggestions, we have reorganized the whole discussion section to reduce unnecessary wording and repetition of ideas (manuscript pages 11-13). We also reduced or removed any new variables or explanations that exceeded the scope of our study. These led to reduction in the number of studies cited, which was also reflected in an updated reference list. We opted to preserve strengths and limitations in paragraph format to not disrupt the flow of information. We additionally included acknowledgements. We added a section on cost-effectiveness as recommended by Reviewer #2.

**RESPONSE TO REVIEWER #2**

**Thanks for the chance to review this study on the diabetes mellitus screening and associated factors in Peru. The study uses data from the 2022 Peruvian National Demographic and Health Survey to inspect the DM screening rates and predictors in this country. The results show a quarter of adult population in Peru had DM screening. Below please find my comments and suggestions on this study.**

**1. Lines 100-114: considering the rich survey data used in this study, it is highly suggested that authors report the weighted prevalence of DM using the ADA criteria to address the updated knowledge on DM prevalence and thereafter enable them to calculate the proportion of undiagnosed DM. This could add significantly to the importance of this study. Also, by adding such statistics, the screening rates could be adjusted for the prevalence of DM to show the real gap on screening.**

We appreciate this suggestion and agree on the importance of determining the proportion of undiagnosed DM in the population. Unfortunately, the Peruvian 2022 DHS did not obtain any laboratory measurements for the surveyed individuals. There is no recorded data on serum glucose levels or HbA1c that would allow us to identify undiagnosed cases of diabetes. We identified screening based on self-report of glucose measurement in the past year. However, we agree with the importance of linking screening coverage with disease prevalence and have reinforced this limitation and its implications for future research in the discussion section.

**2. Results: considering the recently lowered age for DM screening and updated guidelines, the result on the statistically significant odds of screening in 18-34 age group would not infer a clinical importance.**

Thank you for this viewpoint. We apologize for not being clear in our inclusion criteria for this study. The reason we included the population 18-34 in our analysis was because they still meet the current ADA criteria for screening due to obesity and Latino ethnicity. Therefore, we consider the statistically significant less screening prevalence still clinically important as it aligns to current screening recommendations: younger population may be less screened.

**3. Discussion: it is suggested that authors discuss the cost-effectiveness of DM screening in the broader population based on the recent ADA update and considering the shortage of resources in the context of study setting.**

We appreciate this suggestion and have added a brief discussion on the cost-effectiveness of the adoption of this new screening criteria in Peru (manuscript page 13).
