# Supplementary material for: Diabetes Mellitus screening and associated factors in Peru: A cross-sectional analysis of a national health survey
Source: PLOS Glob Public Health. 2025 Sep 3;5(9):e0005150. doi: 10.1371/journal.pgph.0005150 (PMC12407462; doi:10.1371/journal.pgph.0005150)
Supplement: Attachment — Submitted filename: Response_to_Reviewers_auresp_2.docx [file pgph.0005150.s007.docx]

**July 14, 2025**

**Re: Diabetes Mellitus screening and associated factors in Peru: a cross-sectional analysis of a national health survey**

**Einer Carlos Eduardo Arevalo-Rios, M.D., Jennifer Layza-Reyes, M.D., Victor Hugo Noriega-Ruiz, M.D.**

Dear Editorial Board of PLOS Global Public Health,

We are very appreciative of the careful revision for our manuscript. We have addressed each of the points raised in this round of revision. Specifically, changes include additional proofreading of the discussion and minor changes in table format and tone. In the section that follows we have indicated our direct responses to each of the questions raised by your thorough review. In addition, we are attaching the version with tracked changes and an unmarked version of the manuscript. We believe the manuscript is significantly improved because of your observations and hope that you will find the revised version of this manuscript acceptable.

**RESPONSE TO REVIEWER #1**

**General comments:
o please don't use pronouns at all (Like (we))**

Thank you for this feedback. We have switched to use the third person in the article and eliminated first person pronouns from the text.

**o define the abbreviation when used at first time, then use the symbol.**

Thank you for pointing this out. The abbreviations are now defined the first time they are introduced in the article (including abstract). After its definition, only symbols are used.

**o please describe the study population to general idea of the study participants, the author many not need to account the per-centages.**

Thank you for this suggestion. We have implemented this recommendation: the study population section describes a general idea of participants, without excessive percentages. We have also re-titled this section in results to "Characteristics of the study population”, as we noted that “study population” was repeated in the methods section and could lead to confusion (manuscript page 7).

**o better to add variable in the description of study population.**

Thank you for this perspective. We have added variables that were missing in the description of study population, mainly age group, area of residence and ethnicity (manuscript page 7).

**o describe how the author applied the multivariate analysis in the current data. and avoid to write about the results.**

Thank you for this comment. The details of how the multivariate analysis was performed are in the Statistical Analysis subsection of the Methods (manuscript page 6). In the Results section (manuscript page 10), we have revised the text to focus exclusively on reporting the findings from the multivariate analysis, without restating methodological details. We have kept the narrative description of the multivariate analysis results, as we consider the written description important for the readers to understand the most associated factors.

**o inclusion criteria was already mentioned. table 1: characteristics of the population.**

Thank you for this observation, we have removed this from the Table 1 title.

**o it is possible to find bivariate analysis using chi squire test to analyze age ?**

Thank you for this concern. For the bivariate and multivariate analysis, we categorized age as a dichotomous categorical variable based on the ADA screening populations: Younger age 18-34 (1) and 35 and older (2). We used the chi-square test in this situation because we dealt with categorized variables. We have clarified this in Table 2 as “Age (categorized)”.

**o author can use different method for layout to avoid repartition of (ref)**

Thank you for pointing this out. We have adjusted the format of Table 3 to avoid repetition of “Ref”, and now show the Reference category with a Prevalence Ratio (PR) of 1.00.

**o discussion need reread to rewrite in easy, intelligent, have high quality and integrity in language and ideas.**

Thank you for your attention to this. We have proofread the discussion and adapted the language to be precise and easier to read. We hope this is now clearer.

**RESPONSE TO REVIEWER #2**

**Thanks for the revision and responses. I have no further comments.**

Thank you for revising our manuscript.
