# Supplementary material for: Diabetes Mellitus screening and associated factors in Peru: A cross-sectional analysis of a national health survey
Source: PLOS Glob Public Health. 2025 Sep 3;5(9):e0005150. doi: 10.1371/journal.pgph.0005150 (PMC12407462; doi:10.1371/journal.pgph.0005150)
Supplement: Attachment — Submitted filename: Response_to_Reviewers_auresp_3.docx [file pgph.0005150.s009.docx]

**August 8, 2025**

**Re: Diabetes Mellitus screening and associated factors in Peru: a cross-sectional analysis of a national health survey**

**Einer Carlos Eduardo Arevalo-Rios, M.D., Jennifer Layza-Reyes, M.D., Victor Hugo Noriega-Ruiz, M.D.**

Dear Editorial Board of PLOS Global Public Health,

We are very appreciative of this new round of revision. We have addressed the minor changes raised by the reviewer. Specifically, changes include a reorganization of the variables section in ‘Methods’, and minor changes in writing style and tone. In the section that follows we have indicated our direct responses to each of the questions raised. In addition, we are attaching the version with tracked changes and an unmarked version of the manuscript. We hope that you will find the revised version of this manuscript acceptable for publication.

**RESPONSE TO REVIEWER #1**

**General comments:
o Minor revision is recommended.**

Thank you, the minor modifications have been included.

**o All previous comments of the reviewer were not addressed**

We have addressed this round’s comments and those in the attached PDF document.

**o Try to avoid using pronouns (our)**

Thank you for this suggestion. All first-person pronouns, “we”, “our”, “ours” are now removed in the manuscript.

**o Use simple and meanfull language (Recent data from a nationally representative health survey was analyzed to find the prevalence and risk factors associated with lack diabetes screening in Peru)**

Thank you for this perspective. We have revised the ending of the introduction and included this sentence. Unnecessary adjectives were removed from other sections.

**o Inclusion and exclusion criteria better to be with out bulleted or numbering list.**

Thank you for this comment. The inclusion and exclusion criteria are now written in continuous text.

**o The personal and anthropometric and clinical data which obtained in the the questioner are discussed in intelligent way in methods, results and introduction. that mean author must not write the questioner in whole paragraph. And divided it in the paper section according to the related data.**

Thank you for this observation. The ‘Variables’ section was re-written using scientific papers analyzing national surveys as a reference. We chose to preserve this section to communicate our outcome and independent variables to the reader. We have moved the explanation for including altitude of residence to the introduction, and the details of wealth quintiles are now in the results section.

**o *please return to and view scientific papers.**

Thank you. Previous scientific papers have been reviewed and minor corrections have been made in style.

**RESPONSE TO REVIEWER #2**

**The manuscript is well edited. I have no further comments.**

Thank you for this comment.
